# Effect of regional anesthesia and analgesia on long-term survival following abdominal cancer Surgery-A systematic review with meta-analysis

**DOI:** 10.1016/j.heliyon.2023.e20611

**Published:** 2023-10-05

**Authors:** Lin Lu, Yanxia Sun, Yi Ren, Siwen Zhao, Zhen Hua

**Affiliations:** Department of Anesthesiology, Beijing Hospital, National Center of Gerontology, Institute of Geriatric Medicine, Chinese Academy of Medical Sciences, Beijing 100730, China

**Keywords:** regional anesthesia and analgesia, Overall survival, Cancer recurrence, Abdominal cancer

## Abstract

**Background:**

The impact of regional anesthesia and analgesia (RAA) on long-term survival following cancer surgery is a topic of debate. The aim of this study was to investigate the effects of perioperative RAA on long-term oncological outcomes in patients undergoing major abdominal cancer surgery.

**Methods:**

The authors searched computerized databases and reference lists from inception to December 20, 2022. All studies that investigated the effects of perioperative RAA on long-term oncological outcomes following major abdominal cancer surgery were included. Using the inverse variance method with a random-effects model, hazard ratios (HR) and 95% confidence intervals (CI) were calculated.

**Results:**

The systematic review included 51 retrospective studies, one prospective study, and three randomized controlled trials (RCTs), with a total of 95,046 patients. The results showed that perioperative RAA may improve long-term overall survival (HR: 0.85, 95% CI: 0.80 to 0.91, P = 0.00, I^2^ = 60.2%). However, there was no significant association between perioperative RAA and reduced cancer recurrence (HR: 0.98, 95% CI: 0.90 to 1.03, P = 0.31, I^2^ = 52.3%). When performing a pooled analysis of the data from the three RCTs, no statistically significant effect of RAA was found in either case.

**Conclusion:**

The systematic review suggests perioperative RAA may improve long-term overall survival but does not appear to reduce cancer recurrence in patients undergoing major abdominal cancer surgery. The limited number of RCTs included in this study did not confirm this finding, highlighting the need for further RCTs to corroborate these results.

## 1 Introduction

Abdominal cancer refers to the growth of malignant cells in organs and tissues located in the abdominal cavity, which includes the gastrointestinal, urologic, and gynecologic systems. It is a serious and potentially life-threatening condition that affects millions of people worldwide [1]. Although surgical removal of malignant tumors remains the primary treatment option for cancer, many patients still experience recurrence of cancer after surgery, despite advances in surgical techniques and accompanying neoadjuvant and adjuvant therapies [2]. Surgical trauma and perioperative pain are known to induce a stress response, which can impede cell-mediated immunity and promote cancer growth both directly and indirectly [3,4]. In addition, evidence from experimental and animal studies, as well as clinical retrospective studies, suggests that opioid and volatile anesthetics may suppress immune function and contribute to cancer recurrence after surgery [[5], [6], [7]]. Therefore, there is a need to encourage any improvements in anesthetic and pain management.

Regional anesthesia and analgesia (RAA) techniques, including epidural, spinal, and nerve blocks, have been shown to effectively control pain following major abdominal surgery [8]. Studies have demonstrated that RAA can reduce postoperative pain, attenuate the neuro-humoral response to surgical stress, and decrease the need for opioids and intraoperative volatile anesthetics [2,9,10]. These effects of RAA may reduce the risk of cancer recurrence and improve long-term survival after cancer surgery. However, previous systematic reviews examining the effects of RAA on oncological outcomes have produced uncertain results [11,12]. Recently, several large sample-size cohort studies and randomized controlled trials (RCTs) have been published in this topic and the results were inconsistent [[13], [14], [15], [16], [17], [18], [19]]. Therefore, we conducted an up-to-date systematic review and meta-analysis to assess all available evidence on the effects of perioperative RAA on long-term oncological outcomes in adults undergoing abdominal cancer surgery, with the aim of providing additional evidence to guide clinical practice.

## 2 Methods

This systematic review and meta-analysis was presented according to the Preferred Reporting Items for Systematic Reviews and Meta-analyses (PRISMA). A review protocol was written and published in the PROSPERO (CRD 42022358620).

### 2.1 Inclusion and exclusion criteria

The eligible studies of this systematic review and meta-analyses were identified using the patient, intervention, comparison, outcomes, study design (PICOS) strategy.

1) Patients/participants: Adult patients (aged ≥18 years) undergoing abdominal cancer surgery were evaluated. Abdominal cancer surgery was defined as any surgical procedure that involved operating on the abdominal cavity to remove tumors from the gastrointestinal, gynecologic, or urologic systems or any other abdominal cancer. Studies involving pediatric patients or nonsurgical patients were excluded.
2) Type of intervention: RAA used as the intervention for major abdominal cancer surgery which was defined as perioperative administration of regional anesthetics (initiated before surgery, maintained perioperatively, or performed in the immediate postoperative period and lasting up to 6 h after surgery) to provide anesthesia or analgesic effect by the following techniques of RAA: spinal anesthesia (also called subarachnoid block), epidural anesthesia and analgesia, and nerve blocks.
3) Type of comparator: general anesthesia without RAA perioperatively was used as control group. Study which only compared two different types of RAA was not considered in this study.
4) Type of outcomes: Studies in which researchers reported postoperative overall survival or overall mortality or cancer recurrence-free survival or incidence of cancer recurrence or progression-free survival were included. Studies with follow-up period less than 12 months or unknown follow-up were excluded.
5) Type of studies: RCTs and observational study were included. Data from letters, case reports, reviews or abstracts were excluded.

### 2.2 Search strategy and study selection

A systematic search of MEDLINE, EMBASE, Scopus, the Cochrane Controlled Trials Register and Cochrane Database of Systematic Reviews from inception to December 20, 2022, was performed to identify relevant studies using the following search terms: ‘analgesia’ ‘anesthesia’ ‘local’ ‘regional’ ‘spinal’ ‘epidural’ ‘neoplasms’ ‘cancer’ ‘recurrence’ ‘metastasis’ ‘survival’ ‘mortality’ ‘abdomen’ and their Boolean combination. The detailed search strategy is available in Appendix 1 in Supplement 1. No restriction was imposed. The reference lists of all eligible publications and reviews were scanned to identify additional relevant studies. The computer retrieval was supplemented by hand search of the references of relevant original articles and reviews. To identify registered ongoing or unpublished trials, we searched the ClinicalTrials.gov once a month until December 2022.

Two authors (YS and LL) independently screened and reviewed all titles and abstracts for eligibility. For abstracts that did not provide sufficient information to determine eligibility, full-length articles were retrieved. Agreement between the two authors for inclusion of screened articles was measured using weighted kappa [20]. In all steps, any disagreement regarding eligibility was resolved via discussion between the reviewers and a third investigator (ZH) as needed.

### 2.3 Data extraction and quality assessment

Studies were reviewed and data extracted independently by two authors (LL and YS）using a pre-designed standard form with any discrepancy being resolved by reinspection of the original article. Details of perioperative RAA administration were extracted. Other information was also extracted including first author, year of publication, follow-up period, type of cancer and cancer characteristics, total number of patients, details of anesthesia technique, study design, and abdominal procedures.

One of the primary endpoints was cancer recurrence following abdominal cancer surgery which was calculated from operation to recurrence or date of last follow-up. The other primary endpoint was overall survival, which was defined as time from date of surgery to date of death of any cause or date of last follow-up if censored. If five-year data were not available, the longest follow-up data were extracted, otherwise, the five-year follow-up data were used in this study. The risk estimates by maximally adjusted were collected if available. Authors were contacted for detailed information if data was reported only in the figures. If detailed information was not received, the study was excluded from the current meta-analysis.

To assess methodological quality, we used elements of the Newcastle-Ottawa Scale (NOS) for observational studies, including 8 items in following three parts: selection, comparability and outcome (Box1 in Supplement 1) [21]. RCTs were assessed by the Cochrane Collaboration's tool for risk of bias [22]. A study with more than 6 stars of NOS or RCT design was considered as high-quality in this review. The quality of included studies was independently assessed by two authors. Disagreements were settled by consensus or a third reviewer (ZH).

### 2.4 Grading quality of evidence

The quality of evidence for each outcome from different study design was assessed according to Grading of Recommendations, Assessment, Development and Evaluations (GRADE) methods for risk of bias, inconsistency, indirectness, imprecision, and publication bias, and it was evaluated using GRADEPro software 3.6 (GRADE Working Group). These were classified as very low, low, moderate, or high [23].

### 2.5 Statistical analysis

Hazard ratio (HR) with their corresponding confidence interval (CI) value was extracted for analysis. When studies reported both crude and adjusted risk estimates, we used the most fully adjusted estimates. Otherwise, HRs and CIs were extracted from univariate analyses. If HRs were reported without corresponding 95% CIs, the CIs were calculated from the P values provided [24]. The pooled HR were calculated using the inverse variance method with a random-effects model (DerSimonian-Laird estimator). The heterogeneity of the studies was assessed with the Cochran's Q statistics and the I^2^ statistic. Variances of distribution of true proportions among the effects observed in different studies were reported using tau^2^ (τ^2^).

The meta-regression analyses were conducted when appropriate (i.e., number of studies >10) to explore the potential heterogeneity according to type of cancer, methodological quality of included studies and sample size. Pre-specified subgroup analyses were conducted according to types of cancer, study designs (retrospective cohort study, post hoc analysis of previous RCT or RCT), surgical techniques (open or laparoscopic approach) and RAA techniques. Sensitivity analyses were also conducted to test the robustness of the results by excluding the non-high-quality trials and excluding trials without either propensity-score matching or multivariable adjustment. Finally, the influence of each study was evaluated on the overall estimate by calculating random-effects pooled HR omitting each estimate one at a time.

Publication bias was evaluated using contour-enhanced funnel plot in the results involving ≥10 Studies. Egger's test was used to test for asymmetry. In the case of significant asymmetry, Duval and Tweedie's trim and-fill procedure was performed to identify missing studies that should have been plotted. Cumulative meta-analyses by studies' publication time were also conducted for cancer recurrence and overall survival using random effects to identify any trend in the estimates over time. Analyses was conducted using Stata/SE software 16.0 (College Station, TX, USA)

To determine if sufficient data from RCTs were available to draw definitive conclusions, we performed trial sequential analysis (TSA) using TSA version 0.9.5.10 Beta (the Centre for Clinical Intervention Research Department in Copenhagen, Denmark). It's worth noting that TSA analysis was not pre-specified in PROSPERO and should be interpreted as exploratory due to protocol deviation. We calculated the meta-analysis information size required to detect a 10% relative risk reduction in all-cause mortality and cancer recurrence. If the Z-curve crosses one of the predefined boundaries or the optimal sample size line, we can confidently draw definitive conclusions [25]. Inconsistency (I^2^) and diversity (D^2^) were calculated, and results were adjusted for diversity.

## 3 Results

**Fig. 1:**
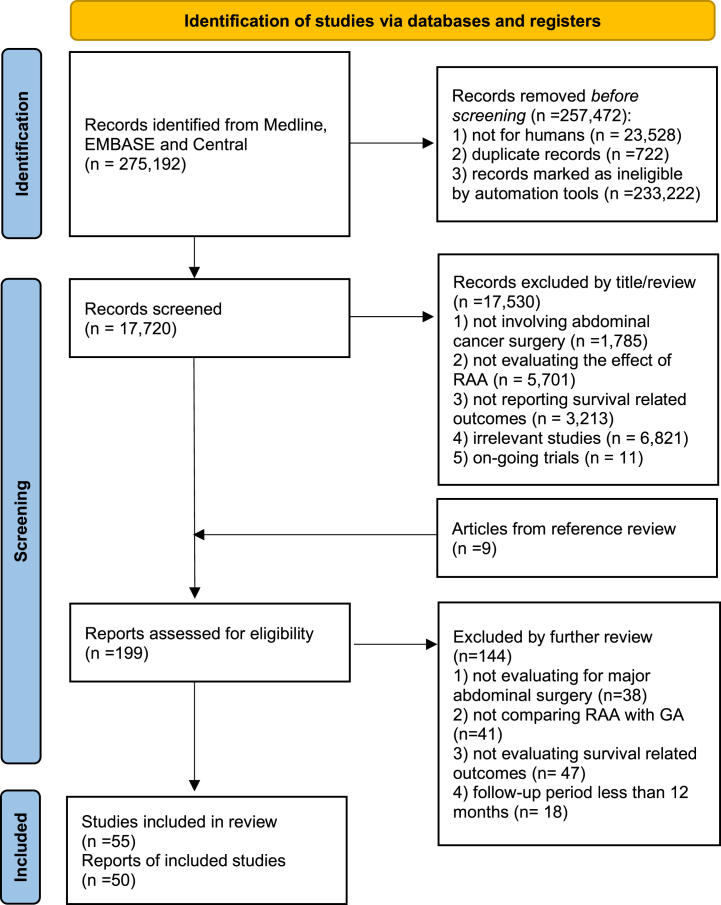
PRISMA flow diagram. RAA, regional anesthesia and analgesia; GA, general anesthesia.

A total of 275,192 records underwent title and abstract screening. Out of these, 256,750 citations were excluded prior to screening due to reasons like not being relevant to humans, duplicate entries, and not meeting eligibility criteria as determined by automated tools. From the remaining 17,720 articles, 17,530 were further excluded as they were not relevant to abdominal cancer surgery, lacked survival-related outcomes, or were ongoing trials. After a detailed evaluation, only 199 articles remained, out of which 144 were further excluded for not meeting specific criteria such as not involving major abdominal surgery or not providing outcomes with a follow-up period of over 12 months. Finally, using the stated eligibility criteria, the authors selected 55 studies unanimously (Fig. 1). In addition, a search for registered ongoing or unpublished trials identified two prospective clinical trials on long-term oncological outcomes following abdominal cancer surgery (see e-Table 1 in Supplement 1).

### 3.1 Description of studies

Fifty-five studies were included [[13], [14], [15], [16], [17], [18], [19],[26], [27], [28], [29], [30], [31], [32], [33], [34], [35], [36], [37], [38], [39], [40], [41], [42], [43], [44], [45], [46], [47], [48], [49], [50], [51], [52], [53], [54], [55], [56], [57], [58], [59], [60], [61], [62], [63], [64], [65], [66], [67], [68], [69], [70], [71], [72], [73]]. All studies were published between 2008 and 2022 in English journals (e-Table 2 in Supplement 1). 51 studies [16,[26], [27], [28], [29], [30], [31], [32], [33], [34], [35], [36], [37], [38], [39], [40], [41], [42], [43], [44], [45], [46], [47], [48], [49], [50], [51], [52], [53], [54], [55], [56], [57], [58], [59], [60], [61], [62], [63], [64], [65], [66], [67], [68], [69], [70], [71], [72], [73]] had retrospective design. Of them, six of them [30,36,39,54,56,66] were *post hoc* analyses of data from previous RCTs which were initially designed for other outcomes. One study was prospective observational study [15] and 3 studies [13,14,19] were RCTs. The NOS of included observational studies ranged from 4 to 9 stars (e-Table 2 and e-Table 3 in Supplement 1). Two of RCTs [13,14] were judged to be low risk and the other one [19] was study with high risk (e-Table 2 and e-Table 4 in Supplement 1). A total of 43 included studies [13,14,16,17,19,27,28,30,31,33,34,[36], [37], [38], [39], [40], [41], [42],^44^,^45^,^47^,^50^,^51^,[54], [55], [56], [57],[59], [60], [61], [62], [63], [64], [65], [66], [67], [68], [69], [70], [71], [72], [73]] were considered as high quality in this systematic review. The kappa statistics showed substantial or perfect agreement between the reviewers (e-Table 5 in Supplement 1).

Nine of the studies included patients with prostate cancer [29,41,59,62,65,66,71,72,74]; 4 studies included patients with bladder cancer [5,34,35,70], one study included patients with renal cancer [17], 16 studies included patients with colorectal cancer [14,16,36,38,39,[42], [43], [44], [45], [46], [47],^49^,^54^,^55^,^63^,^67^,^73^], 8 studies included patients with gastric cancer [37,48,53,57,58,61,68,69], 2 studies included patients with pancreatic cancer [27,51], one study included patients with gallbladder cancer [19], 9 studies included patients with ovarian cancer [18,26,28,32,40,42,50,52,64], 2 studies included patients with hepatocellular carcinoma [31,33], 3 studies included patients with various abdominal malignancies [13,30,56].

### 3.2 Meta-analysis

Five of the identified studies could not be included in the meta-analysis because survival data were presented only in figure format or without figure. One prospective observational study involving 100 bladder cancer patients who underwent radical cystectomy showed that RAA significantly prolonged disease-free survival when compared with general anesthesia [15]. The remaining four studies, which investigated RAA's association with overall survival in patients with ovarian [18], gastric [53,58] and colorectal cancer or cancer recurrence in patients with colorectal cancer [39], found no significant relationship.

#### 3.2.1 Cancer recurrence

**Fig. 2:**
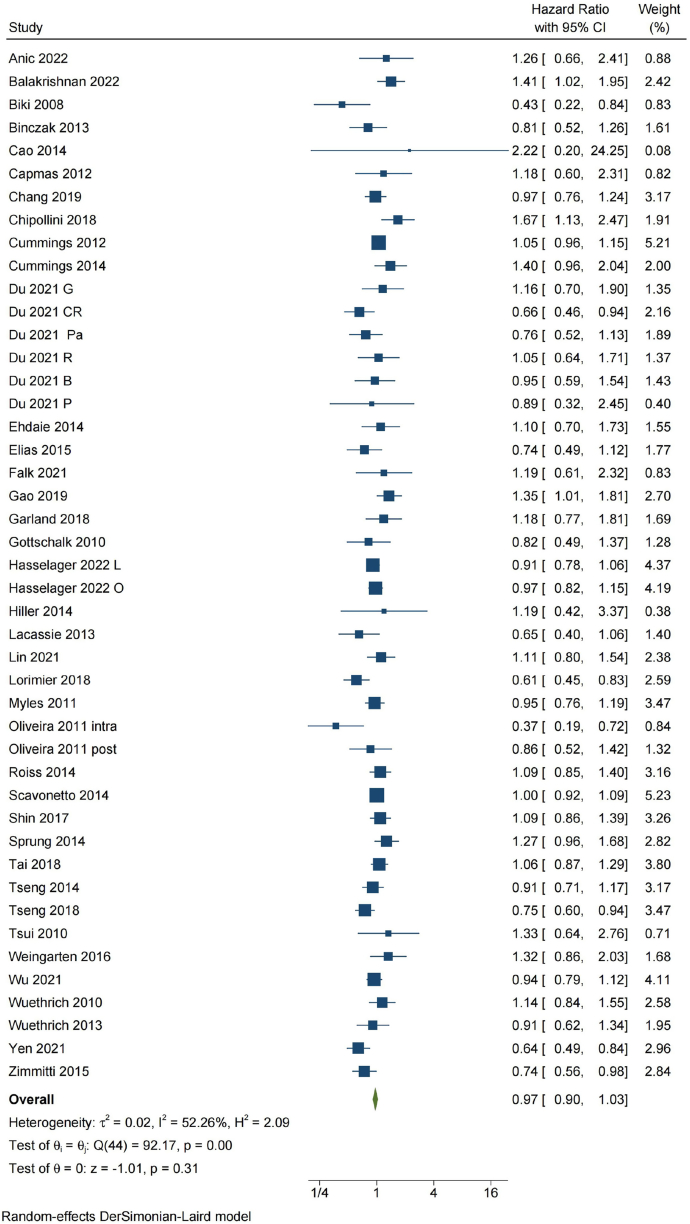
Meta-analysis of the effect of perioperative regional anesthesia and analgesia (RAA) on cancer recurrence after major abdominal cancer surgery. CI, confidence interval; DL, DerSimonian-Laird estimator; G, gastric cancer; CR, colorectal cancer; Pa, pancreatic cancer; R, renal cancer; B, bladder cancer; P, prostate cancer; L, laparoscopy surgery; O, open surgery; intra, epidural anesthesia initiated during surgery; post, epidural anesthesia initiated after surgery.

Thirty-eight studies [13,14,16,17,26,28–34,37,38,40–45,47,48,50,51,54,56,59–66,70–73] with 85,149 patients provided suitable data on cancer recurrence. The unweighted incidence of cancer recurrence in patients who received RAA was 19.8% versus 19.4% in controls. A significant heterogeneity was noted across studies (P = 0.00 for the Q statistics, τ^2^ = 0.02 and I^2^ = 52.3%). Random-effects meta-analysis showed no significant association between RAA and cancer recurrence (HR: 0.97, 95% CI: 0.90 to 1.03, P = 0.31) (Fig. 2).

**Fig. 3:**
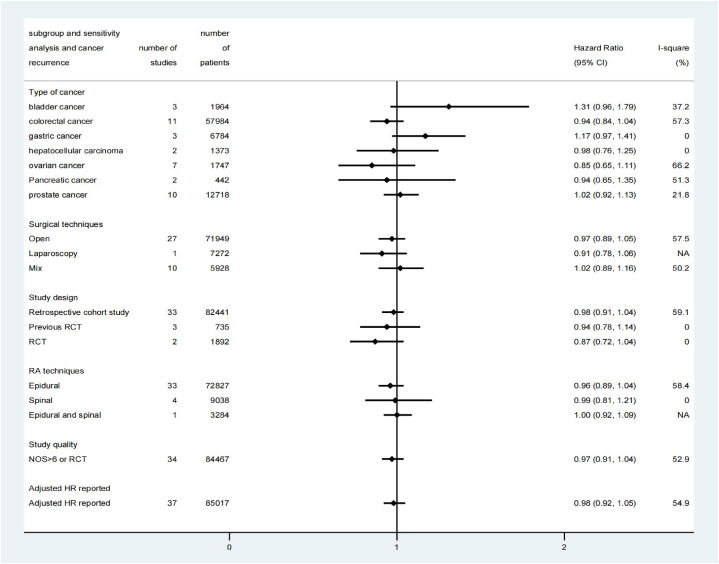
Results of subgroup analyses and sensitivity analyses on cancer recurrence. CI, confidence interval; RCT, randomized controlled trial; NOS, Newcastle-Ottawa Scale; HR, Hazard ratio.

Results of subgroup analyses and sensitivity analyses on cancer recurrence were summarized in Fig. 3. When grouped by different study designs, pooled HR remained no statistically significant in 33 retrospective cohort studies (HR: 0.98, 95% CI, 0.91 to 1.05, P = 0.54, I^2^ = 59.1%), three *post-hoc* analyses of previous RCTs [30,56,66](HR: 0.94, 95% CI, 0.78 to 1.14, P = 0.55, I^2^ = 0) and two RCTs [13,14](HR: 0.87, 95% CI, 0.72 to 1.04, P = 0.13, I^2^ = 0) (Fig. 3 and Figure S1 in Supplement 1). The GRADE quality of evidence from observational data was judged to be very low and the GRADE quality of evidence from RCTs was judged to be moderate (e-Table 6 in Supplement 1).

There was no significant association observed between RAA and cancer recurrence in patients who underwent either open or laparoscopic procedures, or those who received either epidural or spinal techniques (Fig. 3 and Fig. S2 in Supplement 1). When grouped by types of cancer, no significant association was found between RAA and cancer recurrence in any of the included types (Fig. 3 and Figure S3 in Supplement 1). Meta-regression analyses did not reveal a significant effect of all predefined confounders on cancer recurrence (e-Table 7 in Supplement 1). The influence analyses showed each study had no substantial influence on the overall pooled HR (Figure S4 in Supplement 1).

#### 3.2.2 Overall survival

**Fig. 4:**
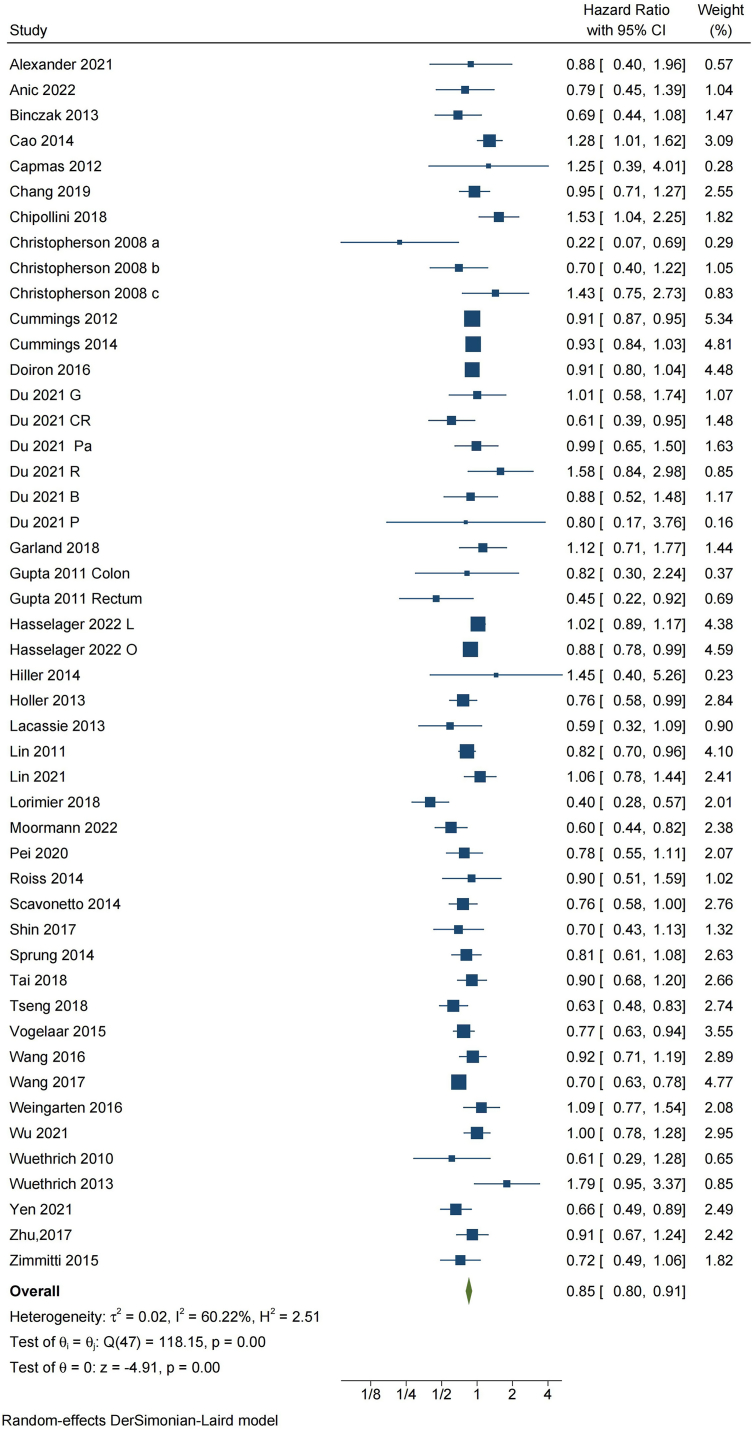
Meta-analysis of the effect of perioperative regional anesthesia and analgesia (RAA) on overall survival after major abdominal cancer surgery. a, nonmetastasis group in first 1.46 years; b, metastasis group; c, nonmetastasis group above 1.46 years; CI, confidence interval; DL, DerSimonian-Laird estimator; Lap, laparoscopy surgery; Open, open surgery; G, gastric cancer; CR, colorectal cancer; Pa, pancreatic cancer; R, renal cancer; B, bladder cancer; P, prostate cancer.

Thirty-nine studies [13,16,17,19,27,28,[30], [31], [32], [33], [34], [35], [36], [37], [38],^44^,[46], [47], [48], [49], [50], [51], [52],^54^,^55^,^57^,[59], [60], [61], [62], [63], [64],[67], [68], [69], [70], [71], [72], [73]] with 91,272 patients provided suitable data about overall survival. The unweighted rate of mortality in patients who received RAA perioperatively was 30.7% versus 38.3% in controls. A significant heterogeneity was noted across studies (P = 0.00 for the Q statistics, τ^2^ = 0.02 and I^2^ = 60.2%). Random-effects meta-analysis showed that RAA was associated with improved overall survival after major abdominal cancer surgery (HR: 0.85, 95% CI: 0.80 to 0.91, P = 0.00) (Fig. 4).

**Fig. 5:**
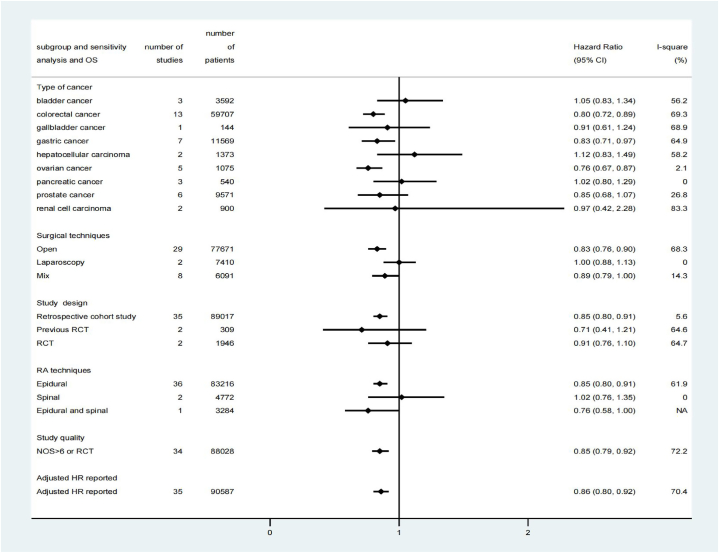
Results of subgroup analyses and sensitivity analyses on overall survival. CI, confidence interval; RCT, randomized controlled trial; NOS: Newcastle-Ottawa Scale; HR, Hazard ratio.

The results of subgroup and sensitivity analyses on overall survival were summarized in Fig. 5. RAA's beneficial effect was observed only in 35 retrospective cohort studies (HR: 0.85; 95% CI, 0.80 to 0.91, P = 0.00, I^2^ = 5.6%) but not in 2 post-hoc analyses of previous RCTs [30,36] (HR: 0.71; 95% CI, 0.41 to 1.22, P = 0.21, I2 = 64.6%) and 2 RCTs [13,19] (HR: 0.91; 95% CI, 0.76 to 1.10, P = 0.33, I2 = 64.7%) (Fig. 5 and Figure S5 in Supplement 1). The GRADE quality of evidence from observational studies was graded as very low, while the quality of evidence from RCTs was rated as low (e-Table 8 in Supplement 1).

In subgroup analyses, RAA showed a significant association with overall survival in patients who underwent open procedures (HR: 0.83; 95% CI, 0.76 to 0.90, P = 0.00, I^2^ = 68.3%) (Fig. 4 and Figure S6 in Supplement 1). Furthermore, statistically significant effects of RAA on overall survival were observed in patients with colorectal cancer [13,16,36,44,46,47,49,54,55,63,67,73] (HR: 0.80; 95% CI, 0.72 to 0.89, P = 0.00, I^2^ = 68.9%), gastric cancer [13,37,48,57,61,68,69] (HR: 0.83; 95% CI, 0.71 to 0.97, P = 0.02, I^2^ = 64.9%), and ovarian cancer [28,32,50,52,66] (HR: 0.76; 95% CI, 0.67 to 0.87, P = 0.00, I^2^ = 2.1%), but not in patients with prostate cancer, bladder cancer, gallbladder cancer, hepatocellular carcinoma, renal cell carcinoma, and pancreatic cancer (Fig. 5 and Figure S7 in Supplement 1). Meta-regression analyses did not reveal any significant effect of the predefined confounders on overall survival (e-Table 7 in Supplement 1). The influence analyses indicated that each study had no substantial influence on the overall pooled HR (Figure S8 in Supplement 1).

### 3.3 Publication bias

Publication bias was assessed for both overall survival and cancer recurrence with the contour enhanced funnel plots shown in Figure S9 in Supplement 1. Egger's test revealed no significant asymmetry in both outcomes (P = 0.36 for overall survival and P = 0.68 for cancer recurrence). The cumulative meta-analysis showed no obvious trend by year of publication on either cancer recurrence or overall survival (Figure S10 in Supplement 1).

### 3.4 Trial sequential analysis for RCTs

We calculated the required information size (RIS) for both all-cause mortality and cancer recurrence. The RIS for all-cause mortality was determined to be 32,170, while the RIS for cancer recurrence was found to be 24,391. It is worth noting that the cumulative Z curve depicted in Fig. S11 in Supplement 1 has not yet reached RIS either for all-cause mortality or cancer recurrence. TSA showed 35% I^2^ and 49% D^2^ with a pooled effect of 0.98 (CI adjusted by diversity: 0.65 to 1.49) for cancer recurrence. For all-cause mortality, TSA indicated an I^2^ of 80% and D^2^ of 85%, with a pooled effect of 0.92 (CI adjusted by diversity: 0.53 to 1.59).

## 4 Discussion

The systematic review's findings suggest that perioperative RAA may improve overall survival in patients undergoing abdominal cancer surgery. However, no significant evidence supports the association between RAA and a reduction in cancer recurrence following abdominal cancer.

RAA has been shown to offer several potential benefits for patients undergoing major abdominal cancer surgery, which have led to a hypothesis that RAA may improve long-term survival following cancer surgery. However, the majority of published studies were retrospective in design, and the results were conflicting. The previous systematic review [11] based on retrospective data indicated that RAA had a positive impact on the overall survival of patients who underwent cancer resection surgery but did not show significant effects on cancer recurrence. However, these findings were based on retrospective data and did not necessarily imply a causal link. In a recent meta-analysis of six RCTs [12], including two breast cancers and four abdominal cancers, found no significant difference in cancer recurrence and all-cause mortality between patients who received RAA and those who did not. However, it should be noted that all four studies that focused on abdominal cancers in this review were post hoc analyses of data from previous RCTs and not prospective RCTs, meaning the evidence presented in this systematic review about abdominal cancer was based on studies with a retrospective design. The current systematic review is the most up-to-date analysis of the effects of RAA on long-term oncological outcomes following abdominal cancer surgery, with a comprehensive search strategy that included new large sample-size observational studies [16,47,69], one prospective study [15] and three prospective RCTs [13,14,19]. The findings of this study align with a previous meta-analysis [11]that showed a significant association between RAA and improved survival in patients who underwent abdominal cancer surgery. However, this study did not find evidence of an association between RAA and cancer recurrence. The discrepancy between overall and recurrence-free survival may have several explanations. Experimental studies suggest that although RAA may not directly impact recurrence, it may slow down cancer progression, resulting in longer survival [74]. RAA may also increase survival through means unrelated to the underlying disease, such as better rehabilitation, reduced chronic pain, and anti-inflammatory properties [56]. However, it's crucial to mention that RAA didn't demonstrate any connection with enhanced overall survival or reduced cancer recurrence when restricted analysis for three RCTs. The TSA findings suggest that the available data is insufficient to establish conclusive correlations between RAA and long-term oncological outcomes. Although the TSA analysis was exploratory in nature, it emphasized the current inability to draw definitive conclusions from the RCT and highlighted the crucial need for additional research.

It is important to note that current evidence only assesses the impact of epidural and spinal anesthesia and analgesia on long-term outcomes. The efficacy of these RAA techniques in laparoscopic procedures is controversial, as postoperative pain management may eliminate the need for epidurals. With limited evidence available on the use of RAA in laparoscopic procedures, only three studies were included, and no benefits of RAA were found. Therefore, it is crucial to explore and reassess alternative analgesic strategies, such as nerve blocks, to improve patient outcomes and promote long-term recovery in laparoscopic procedures.

It is essential to consider the limitations of this review when interpreting the results. Most included studies were retrospective and could introduce biases and confounding factors. Additionally, with only three RCTs, it is premature to draw definitive conclusions. Despite conducting meta-regression analyses, certain confounders were not significant, and heterogeneity resulted from the inability to standardize confounders across all studies. The RAA strategy also varied significantly, leading to different physiologic impacts and difficulties in determining its effect on oncological outcomes. Although no statistically significant difference was found in cancer recurrence, it is challenging to draw a definitive conclusion regarding RAA's effect. Finally, publication bias cannot be excluded, even though the Egger test showed no evidence of it and cumulative analyses did not indicate any temporal effect.

## 5 Conclusions

This systematic review suggests that perioperative RAA may improve overall survival but does not seem to reduce the risk of cancer recurrence following abdominal cancer surgery. Nonetheless, evidence from a limited number of RCTs does not conclusively demonstrate the advantages of RAA on cancer recurrence and overall survival. Thus, to elucidate this critical issue, further large-scale, well-designed prospective RCTs are imperative.

## Funding

None.

## Data availability statement

Data will be made available on request.

## CRediT authorship contribution statement

**Lin Lu:** Conceptualization, Data curation, Formal analysis, Methodology, Project administration, Writing – original draft, Writing – review & editing. **Yanxia Sun:** Conceptualization, Data curation, Formal analysis, Investigation, Methodology, Project administration, Supervision, Validation, Writing – original draft, Writing – review & editing. **Yi Ren:** Data curation, Formal analysis, Writing – original draft. **Siwen Zhao:** Conceptualization, Data curation, Software, Writing – original draft. **Zhen Hua:** Conceptualization, Data curation, Methodology, Resources, Validation, Visualization, Writing – review & editing.

## Declaration of competing interest

The authors declare that they have no known competing financial interests or personal relationships that could have appeared to influence the work reported in this paper.
