# Supplementary material for: Effect of regional anesthesia and analgesia on long-term survival following abdominal cancer Surgery-A systematic review with meta-analysis
Source: Heliyon. 2023 Oct 5;9(10):e20611. doi: 10.1016/j.heliyon.2023.e20611 (PMC10570603; doi:10.1016/j.heliyon.2023.e20611)
Supplement: Multimedia component 1 [file mmc1.docx]

All data relevant to the study are included in the article or uploaded as supplemental information. Our search protocol is available on PROSPERO (CRD 42022358620) and all search strategies are available in supplemental materials.
