# Supplementary material for: Effect of regional anesthesia and analgesia on long-term survival following abdominal cancer Surgery-A systematic review with meta-analysis
Source: Heliyon. 2023 Oct 5;9(10):e20611. doi: 10.1016/j.heliyon.2023.e20611 (PMC10570603; doi:10.1016/j.heliyon.2023.e20611)

Appendix 1 search strategy

Box 1: Elements of the Newcastle-Ottawa Scale (NOS)

e-Table 1: Registered ongoing or unpublished prospective clinical trials

e-Table 2: Characteristics of the 55 Included Studies

e-Table 3: Cohort studies assessed by Newcastle-Ottawa Scale (NOS)

e-Table 4：RCTs assessed by Cochrane Collaboration’s tool. RCTS, randomized controlled trials.

e-Table 5: Weighted Kappa Measurements to Assess Agreement Between Reviewers in Rating Quality of Methodology of Included Trials

e-Table 6: Grade quality of evidence for cancer recurrence

e-Table 7: Meta-regression analyses for cancer recurrence and overall survival

e-Table 8: Grade quality of evidence for overall survival

Figure S1: RAA and cancer recurrence: Forrest plots of subgroup analysis based on study design for cancer recurrence

Figure S2: RAA and cancer recurrence: Forrest plots of subgroup analysis based on RAA techniques and surgical techniques

Figure S3: RAA and cancer recurrence: Forrest plots of subgroup analysis based on types of cancer.

Figure S4：RAA and cancer recurrence: Leave-one-out meta-analysis

Figure S5: RAA and overall survival: Forrest plots of subgroup analysis based on study design for cancer recurrence.

Figure S6: RAA and overall survival: Forrest plots of subgroup analysis based on RAA techniques and surgical techniques.

Figure S7: RAA and overall survival: Forrest plots of subgroup analysis based on types of cancer.

Figure S8: RAA and overall survival: Leave-one-out meta-analysis

Figure S9: Contour-enhanced funnel plots

Figure S10: Cumulative meta-analysis by publication year for cancer recurrence (left) and overall survival (right)

Figure S11: Trial sequential analyses of RCTs

**Supplementary Appendix 1: Search strategy**

**MEDLINE search strategy**

exp analgesia

exp anesthesia

exp regional

exp local

exp locoregional

exp epidural

exp spinal

exp subarachnoid block

exp nerve block

exp peripheral

exp regional anesthetics

exp local agents

exp analgesic effect

exp anesthetic technique

1 or 2 or 3 or 4 or 5 or 6 or 7 or 8 or 9 or 10 or 11 or 12 or 13 or 14

exp neoplasm

exp cancer

exp tumor

exp carcinoma

exp metastasis

16 or 17 or 18 or 19 or 20

exp recurrence

exp survival

exp survival analyses

exp mortality

exp death

exp prognosis

exp prognosis analyses

exp disease-free survival

exp progression-free survival

exp Kaplan-Meier survival

exp cancer-free survival

exp biochemical recurrence

exp cancer recurrence-free survival

exp biochemical recurrence-free survival

exp recurrence-free survival

exp cancer-specific survival

exp disease-free survival

exp postoperative progression-free survival

22 or 23 or 24 or 25 or 26 or 27 or 28 or 29 or 30 or 31 or 32 or 33 or 34 or 35 or 36 or 37 or 38 or 39

exp abdomen

exp abdominal

exp abdominal procedures

exp abdominal cancer surgery

exp cancer surgery

exp carcinoma Surgery

exp gastrointestinal

exp stomach

exp gastric

exp gastrostomy

exp bladder

exp cystectomy

exp hepatocellular

exp pancreatic

exp pancreatectomy

exp ovarian

exp cytoreduction

exp colorectal

exp rectal

exp colon

exp colectomy

exp hepatic resection

exp renal cell carcinoma

exp gallbladder

exp prostate

exp prostatectomy

exp abdominal malignancies

41 or 42 or 43 or 44 or 45 or 46 or 47 or 48 or 49 or 50 or 51 or 52 or 53 or 54 or 55 or 56 or 57 or 58 or 59 or 60 or 61 or 62 or 63 or 64 or 65 or 66 or 67

15 and 21 and 40 and 68

**EMBASE search strategy:**

('regional':ti,ab OR 'epidural':ti,ab OR 'never':ti,ab OR 'pain':ti,ab OR 'spinal':ti,ab OR subarachnoid ':ti,ab OR 'block':ti,ab) AND ('neoplasm'/exp OR 'acral tumor':ti,ab OR 'acral tumour':ti,ab OR 'neoplasia':ti,ab OR 'neoplasm':ti,ab OR 'neoplasms':ti,ab OR 'neoplasms by histologic type':ti,ab OR 'neoplasms, cystic, mucinous, and serous':ti,ab OR 'neoplasms, embryonal and mixed':ti,ab OR 'neoplasms, germ cell and embryonal':ti,ab OR 'neoplasms, glandular and epithelial':ti,ab OR 'neoplasms, hormone-dependent':ti,ab OR 'neoplasms, post-traumatic':ti,ab OR 'neoplastic disease':ti,ab OR 'tumor':ti,ab OR 'tumour':ti,ab OR 'malignant neoplasm'/exp OR 'cancer':ti,ab OR 'cancers':ti,ab OR 'malignant neoplasia':ti,ab OR 'malignant neoplasm':ti,ab OR 'malignant neoplastic disease':ti,ab OR 'malignant tumor':ti,ab OR 'malignant tumour':ti,ab OR 'neoplasia, malignant':ti,ab OR 'tumor, malignant':ti,ab OR 'tumour, malignant':ti,ab) AND (long-term survival OR long-term outcomes OR long-term effects OR long-term prognosis) ('survival rate'/exp OR 'rate, survival':ti,ab OR 'survival curve':ti,ab OR 'survival probability':ti,ab OR 'survival rate':ti,ab OR 'recurrence free survival'/exp OR 'rfs (recurrence free suvival)':ti,ab OR 'recurrence free survival':ti,ab OR 'relapse free survival':ti,ab OR 'all cause mortality'/exp OR 'all cause mortality':ti,ab OR 'overall survival'/exp OR 'overall survival':ti,ab OR 'kaplan meier method'/exp OR 'kaplan meier analysis':ti,ab OR 'kaplan meier curve':ti,ab OR 'kaplan meier estimate':ti,ab OR 'kaplan meier estimation':ti,ab OR 'kaplan meier estimator':ti,ab OR 'kaplan meier method':ti,ab OR 'kaplan meier survival curve':ti,ab OR 'kaplan meiers estimate':ti,ab OR 'kaplan-meier analysis':ti,ab OR 'kaplan-meier curve':ti,ab OR 'kaplan-meier estimate':ti,ab OR 'kaplan-meier estimation':ti,ab OR 'kaplan-meier estimator':ti,ab OR 'kaplan-meier method':ti,ab OR 'kaplan-meier survival curve':ti,ab OR 'kaplan-meiers estimate':ti,ab OR 'cox proportional hazards model'/exp OR 'all-cause mortality' OR 'recurrence-free survival' OR 'overall survival')

Refined by: [excluding] DOCUMENT TYPES: ( EDITORIAL MATERIAL OR REVIEW OR LETTER ) AND [excluding] WEB OF SCIENCE CATEGORIES: ( VETERINARY SCIENCES )

**Scopus Strategy:**

TITLE-ABS-KEY ( ( "neoplasia" OR "neoplasias" OR "neoplasm" OR "tumor" OR "tumors" OR "tumour" OR "tumours" OR "cancer" OR "cancers" OR "malignancy" OR "malignancies" OR "malignant neoplasm" OR "malignant neoplasms" ) AND ("regional" OR "epidural" OR "spinal" OR "nerve block" OR "nerve blocks" OR "spinal" OR " subarachnoid " OR "pain") AND ( "mortality" OR "all-cause mortality" OR "survival" OR "overall survival" OR "Kaplan-meier" OR "proportional hazard model" OR "cox regression" OR "recurrence-free survival" OR "recurrence" OR "local recurrence" ) ) AND ( EXCLUDE ( DOCTYPE , "re" ) OR EXCLUDE ( DOCTYPE , "le" ) OR EXCLUDE ( DOCTYPE , "ed" ) OR EXCLUDE ( DOCTYPE , "cp" ) OR EXCLUDE ( DOCTYPE , "no" ) OR EXCLUDE ( DOCTYPE , "sh" ) OR EXCLUDE ( DOCTYPE , "ch" ) OR EXCLUDE ( DOCTYPE , "er" ) ) AND ( EXCLUDE ( SUBJAREA , "VETE" ) )

**Cochrane Library search strategy**

ID Search Hits

#1 MeSH descriptor: [Neoplasms] explode all trees 76186

#2 MeSH descriptor: [Neoplasms] explode all trees and with qualifier(s): [mortality - MO, surgery - SU, complications - CO] 24050

#3 “neoplasia” 2918

#4 neoplasias 469

#5 neoplasm 24598

#6 tumor 61364

#7 tumors 21826

#8 tumour 61333

#9 tumours 21825

#10 cancer 172351

#11 cancers 10753

#12 malignancy 6660

#13 malignancies 6264

#14 “malignant neoplasm” 3448

#15 “malignant neoplasms” 382

#16 {OR #1-#15} 214743

#17 MeSH descriptor: [Mortality] explode all trees 12664

#18 MeSH descriptor: [Mortality] explode all trees 12664

#19 MeSH descriptor: [Survival Rate] explode all trees 9570

#20 MeSH descriptor: [Survival Analysis] explode all trees 19898

#21 MeSH descriptor: [Kaplan-Meier Estimate] explode all trees 4817

#22 MeSH descriptor: [Proportional Hazards Models] explode all trees 4876

#23 MeSH descriptor: [Neoplasm Recurrence, Local] explode all trees 4016

#24 MeSH descriptor: [Recurrence] explode all trees 11814

#25 mortality 90386

#26 "all-cause mortality" 8790

#27 survival 102357

#28 "overall survival" 37550

#29 "kaplan-meier" 12933

#30 "proportional hazard model" 546

#31 "cox regression" 4836

#32 "recurrence-free survival" 2518

#33 "recurrence" 47010

#34 "local recurrence" 2137

#35 {OR #16-#34} 195911

#36 MeSH descriptor: [Analgesia, Epidural] explode all trees 2226

#37 MeSH descriptor: [Anesthesia, Spinal] explode all trees 2608

#38MeSH descriptor: [Nerve Block] explode all trees 4998

#39 MeSH descriptor: [Pain Management] explode all trees 4989

#40 (spinal):ti,ab,kw 32641

#41 (epidural):ti,ab,kw 14320

#42 ("nerve block anesthesia"):ti,ab,kw 141

#43 ("regional anesthesia"):ti,ab,kw 3801

#44 {OR #36-#43} 101128

#45#16 AND #35 3321

#46 #16 AND #35 AND #44 243

**BOX 1: NEWCASTLE - OTTAWA QUALITY ASSESSMENT SCALE COHORT STUDIES**

Note: A study can be awarded a maximum of one star for each numbered item within the Selection and Outcome categories. A maximum of two stars can be given for Comparability

Selection

1) Representativeness of the exposed cohort

a) Truly representative of the average ____incidence of cancer____in the community ****

b) Somewhat representative of the average ___incidence of cancer__in the community ****

c) Selected group of users eg nurses, volunteers

d) No description of the derivation of the cohort

2) Selection of the non exposed cohort

a) Drawn from the same community as the exposed cohort ****

b) Drawn from a different source

c) No description of the derivation of the non exposed cohort

3) Ascertainment of exposure

a) Secure record (eg surgical records) ****

b) Structured interview 

c) Written self report

d) No description

4) Demonstration that outcome of interest was not present at start of study

a) Yes ****

b) No

Comparability

1) Comparability of cohorts on the basis of the design or analysis

a) Study controls for _____age________ ****

b) Study controls for any additional factors: tumour stage, transfusion, blood loss** **

Outcome

1) Assessment of outcome

a) Independent blind assessment ****

b) Record linkage ****

c) Self report

d) No description

2) Was follow-up long enough for outcomes to occur

a) Yes (more than 3 years) ****

b) No

3) Adequacy of follow up of cohorts

a) Complete follow up - all subjects accounted for  ****

b) Subjects lost to follow up unlikely to introduce bias - small number lost follow up rate > _70___ % ****

c) Follow up rate < _70___% and no description of those lost

d) No statement

**e-Table 1: Registered ongoing or unpublished prospective clinical trials on abdominal cancer**

| Registration number | Title | Type of cancer |
| --- | --- | --- |
| NCT02681796 | Epidural Analgesia Use in Pancreatic Resections | - Pancreas Cancer |
| NCT01318161 | Epidural Versus Patient-controlled Analgesia for Reduction in Long-term Mortality Following Colorectal Cancer Surgery | - Colorectal Cancer |

**e-Table 2: The characteristics of included studies**

| Trials | Date of surgery | Follow-up period | NOS score | Number of patients | Type of tumor | Anesthetic and pain management | | Study design | Type of procedure |
| --- | --- | --- | --- | --- | --- | --- | --- | --- | --- |
|  |  |  |  |  |  | Intervention (n): details of RAA strategy | Control (n) |  |  |
| Alexander 2021 [27] | NA | 5y/17.26m(median) | 7 | 98 | Pancreatic cancer | GA+EDA (70): at T6-T10,15–150 mg 0.375–0.75% ropivacaine initially, 0.375% ropivacaine 7.5–50 mg/h + sufentanil (total dose 15–135 µg) according to anesthesiologist during surgery, 0.2% ropivacaine 4–12 mL/h postoperatively | GA+PCIA (28) | Retrospective cohort study | Partial pancreatoduodenectomy, distal and total pancreatectomy |
| Anic 2022 [28] | 2008.1-2019.12 | 11.5 y | 7 | 110 | Ovarian cancer | GA+EDA (71): bupivacaine 0,25% or ropivacaine 0,375% with or without sufentanil intra-operatively, bupivacaine 0,125% alone in the normal ward and combined fentanyl in intensive or intermediate care units postoperatively | GA+PCIA (39) | Retrospective cohort study | Tumor debulking surgery |
| Balakrishnan 2022 [26] | 2013-2017 | 96m^1^ | 7 | 312 | Epithelial ovarian tumors | GA+EDA (196): at lower thoracic levels, bupivacaine 0.1-0.125% with morphine intra and post -operatively for 72 h | GA+IV paracetamol and tramadol postoperatively (116) | Retrospective cohort study | Elective optimal cytoreduction |
| Biki 2008 [29] | 1994.1-2003.12 | 2.8-12.8y | 6 | 225 | Prostate cancer | GA+EDA (102): at thoracic spine (T11–T12), initiated before surgery and continued for 48-72h postoperatively | GA+ PCIA (39) | Retrospective cohort study | Radical prostatectomy |
| Binczak M 2013 [30] | 1990.2-1991.4 | 17.3y (median) | 7 | 132 | Various abdominal malignancies | GA+EDA (69): at T7-T11, 20 ml bolus of 0.25% bupivacaine before surgery and 0.125% bupivacaine (10 ml/h) + morphine (0.25 mg/h) lasted until the 5^th^ postoperative day | GA+ subcutaneous morphine(2.5mg/) postoperatively (63) | Previous RCT^2^ | Major abdominal surgery for cancer |
| Cao 2014 [31] | 1997.1–2007.12 | 2-13y | 7 | 819 | hepatocellular carcinoma | GA+EDA (451):at T9-T10 or T10-T11, 0.33% ropivacaine+2 mg morphine 6 mL bolus at the time of fascial closure, 0.15% ropivacaine +0.07 mg/kg per day morphine at rate of 2 mL/h for 48 hours after surgery | GA +PCIA (368) | Retrospective cohort study | Hepatic resection |
| Capmas 2012 [32] | 2007.1-2009.12 | 45m^1^ | 6 | 94 | Ovarian cancer | GA+ EDA(47): at T9-T12, bolus of 0.2% ropivacaine (20-25 ml) 45 to 60 mins before end of surgery, postop PCEA with 0.2% ropivacaine 3 to 6 ml/h and bolus of 3 ml with blockout period of 20 minutes for 3 days, morphine (5 mg) added to every 200 ml vial of ropivacaine | GA (47) | Retrospective cohort study | Complete cytoreduction |
| Chang 2019 [33] | 2005.1-2011.12 | 150m^1^ | 8 | 554 | Hepatocellular carcinoma | GA+EDA (435): at lower thoracic spine, initiated 1 day before surgery and lasted 48 to 72h after surgery with 0.25%- 0.5 % bupivacaine at rate of 5-10 mL/h | GA+PCIA (63) | Retrospective cohort study | Hepatocellular carcinoma resection |
| Chipollin 2018 [34] | 2008.2-2012.8 | 0.2-101m/41.4m(median) | 7 | 430 | Bladder cancer | GA+EDA (215): at T10-T12, sufentanil (median intraoperative dose =50mcg, range 45–85) during intra and postoperative period | GA+ IV morphine or hydromorphone postoperatively (215) | Retrospective cohort study | Radical cystectomy with pelvic lymph node dissection |
| Christopherson 2008 [36] | 1992.3-1994.8 | 8.3-10.8y | 7 | 177 | Colon cancer | GA+EDA (85): at lumbar or thoracic level, initiated before surgery and lasted as long as judged clinically appropriate after surgery with boluses of 5–10 mL 0.5% bupivacaine with or without morphine and other drugs | GA + IV opioids postoperatively (92) | Previous RCT^2^ | Colectomy |
| Cummings 2012 [38] | 1996-2005 | EDA 5.3y; GA 4.8y (median) | 8 | 42151 | Colorectal cancer | GA+EDA (9670): at lumbar or thoracic level, perioperatively | GA (32481) | Retrospective cohort study | Colectomy |
| Cummings 2014 [37] | 1996–2005 | 160m^1^ | 8 | 2745 | Non-metastatic gastric carcinoma | EDA (766): at lumbar or thoracic level, perioperatively | GA (1979) | Retrospective cohort study | Gastrectomy |
| de Oliveira 2011 [40] | 2000.1-2006.10 | 42m (median) | 7 | 182 | Ovarian cancer | GA+ postop EDA (29)/GA+ EDA (26):at T10-T12 level, intra EDA with bupivacaine 10mg bolus and an infusion of 12.5 mg/hr, postop EDA with bupivacaine 1 mg/mL plus hydromorphone 10Kg/mL(continuous 4 mL/h, patient-controlled bolus 4mL) for 48 to 72 h | GA+PCIA (127) | Retrospective cohort study | Debulking surgery for ovarian cancer |
| Day 2012 [39] | 2003.10-2010.12 | EDA 37m; PCA 28m (median) | 7 | 424 | Colorectal cancer | GA+EDA (107)/ GA+SPA (144): EDA at T9 and T12, with 10 ml bupivacaine 0.2% + fentanyl 100 mg bolus, continue with bupivacaine 0.15% +fentanyl 0.0002% at 4–8 ml/h, lasted 48h after surgery; SPA at L2–3 or L3–4, with 2.5 ml hyperbaric bupivacaine 0.5%+diamorphine 0.25 mg before induction | GA+PCIA (173) | Previous RCT^2^ | Laparoscopic colorectal resection |
| Doiron 2016 [35] | 2004-2008 | 5 y | 6 | 1628 | Bladder cancer | GA+EDA (887): at thoracic level perioperatively | GA (741) | Retrospective cohort study | Radical cystectomy |
| Du 2019 [13] | 2011.11.21-2015.5.25 | 4-8y/ 66m (median) | Low risk ^4^ | 1802 | Abdominal cancer 67.9%^3^ | GA+EDA (853): 0.375 to 0.5% ropivacaine during surgery, 0.12% ropivacaine+0.5 μg/ml sufentanil 4 ml/h and 2ml bolus dose with lockout time 20min postoperatively | GA+PCIA (859) | Randomized multicenter controlled trial | Major noncardiac thoracic and abdominal surgery |
| Ehdaie 2014 [41] | 1999-2008 | 4.6 y | 9 | 929 | Prostate cancer | SPA (264): SPA in L3/4 or L4/5, with 0.5% bupivacaine 10 mg + epinephrine 0.1 mL 1:200,000, sedation with propofol + 25-50ug fentanyl intraoperatively | GA+ PCIA (665) | Retrospective cohort study | Radical prostatectomy |
| Elias 2015 [42] | 2007.1.1-2011.12.31 | 46 m (median) | 7 | 194 | Stage III epithelial ovarian cancer | GA+PCEA (60): bupivacaine with or without hydromorphone postoperatively | GA (134) | Retrospective cohort study | Primary cytoreductive surgery |
| Falk 2021 [14] | 2011.6-2017.5 | 5 y | Low risk ^4^ | 180 | Colorectal cancer | GA+EDA (89):at T10-T12, using combination of local anesthetic and opioid according to local hospital routines, for 72 h postoperatively | GA +PCIA (91) | Randomized multicenter controlled trial | Open or minimally invasive curative colorectal cancer surgery |
| Gao 2019 [43] | 2007.5-2012.7 | 5-10y | 6 | 225 | Colorectal carcinoma liver metastases | GA+EDA (92): at T7-T10, with 7-10 ml 0.75% ropivacaine bolus followed by 0.2% ropivacaine 6 ml/h, initiated before surgery and stopped after surgery | GA (133) | Retrospective cohort study | Colorectal carcinoma liver metastasis resection |
| Garland 2018 [44] | 1996-2015 | 5 y | 7 | 172 | Colorectal carcinoma liver metastases | GA+EDA (44): at thoracic level, with typically local anesthetics (bupivacaine and/or hydromorphone) | GA +PCIA (128) | Retrospective cohort study | Hepatic resection |
| Gottschalk 2010 [45] | 2000.1-2007.3 | 1.8y (median) | 7 | 256 | Colorectal cancer | GA+ EDA (256): EDA perioperatively | GA (253) | Retrospective cohort study | Open colectomy |
| Gupta 2011 [46] | 2004.1-2008.1 | 1-5y | 6 | 655 | Colorectal cancer | GA+ EDA (562): LA with or without fentanyl perioperatively and LA with fentanyl or morphine for 2-5 days postoperatively | GA+PCIA (128) | Retrospective cohort study | Open colectomy |
| Hasselager 2022 [47] | 2004-2018.9 | 58 m | 9 | 11618 | Colorectal cancer | GA+EDA (laparotomy1672; laparoscopy1824): initiated before surgery | GA (laparotomy2674; laparoscopy5448) | Retrospective cohort Study | Colorectal cancer resections |
| Hiller 2014 [48] | 2005.1-2010.1 | 2y | 5 | 140 (Esophageal 71; Gastric 69) | Gastro-esophageal cancer | GA+EDA (97): at thoracic level, 0.5% bupivacaine 3–5 ml repeated intra-operatively, 0.125% bupivacaine 6–12 ml/h+2–3 mg diamorphine bolus daily postoperatively | GA+ morphine or fentanyl postoperatively (43) | Retrospective cohort study | Gastro-oesophageal cancer surgery |
| Holler 2013 [49] | 2003.1-2009.12 | 1-8y | 5 | 749 | Colorectal cancer | GA+EDA (442): initiated before surgery and for postoperative pain management as clinically indicated | GA (307) | Retrospective cohort study | Colorectal cancer resections |
| Lacassie 2013 [50] | 2000.1 -2011.3 | 4.9y (median) | 9 | 80 | Advanced ovarian cancer | GA+EDA (37): at T10-L1, intermittent bolus with bupivacaine 0.1%–0.5% with or without fentanyl during surgery, local anesthetics +opioids via EDA for at least 48h postoperatively | GA +PCIA (43) | Retrospective cohort study | Exploratory laparotomy for  ovarian cancer |
| Lin 2011 [51] | 1994.1-2006.10 | 2.0-14.5y | 6 | 143 | Ovarian serous adenocarcinoma | EDA (106): at T11–T12 or L2–L3, continued with bupivacaine 0.125% or ropivacaine 0.150%+ morphine 6–8 mg until 48h after surgery | GA+PCIA (37) | Retrospective cohort study | Laparotomy for ovarian carcinoma |
| Lin 2021 [52] | 2008.1-2017.12 | 15.9 m (median) | 8 | 252 | Pancreatic cancer | GA+EDA (88):at T9-T11, 150 mg xylocaine +50 µg fentanyl for bolus, 0.25% bupivacaine 5-10 ml/h intraoperatively, lasted for 48-72h postoperatively | GA+IV morphine postoperatively (164) | Retrospective cohort study | Resection of pancreatic cancer |
| Liu 2021 [53] | NA | 3y | 4 | 200 | Gastric cancer | GA+EDA (100): at L1-L2 ropivacaine (0.7%) 6 – 8 ml after induction, 0.7% ropivacaine 5ml every 1 h during surgery, PCIA with morphine (0.5- 2.0mg bolus, lockout time 5 min) postoperatively | GA+PCIA (100) | Retrospective cohort study | Laparoscopic-assisted gastric tumor resection |
| Lorimier 2018 [54] | 2005-2016 | 46.1m (median) | 8 | 150 | Colorectal peritoneal metastases | GA+EDA (91): at T7-T8 or T8-T9, 0.2% ropivacaine + sulfentanil 1 mg/ml at 2-10 ml/h, lasted 2 to 7 days postoperatively | GA+PCIA (59) | Previous RCT^2^ | Complete cytoreductive surgery and hyperthermic intraperitoneal chemotherapy |
| Moormann 2022 [55] | 1999-2007 | 100m^1^ | 7 | 215 | Colon cancer | GA+EDA (83):at thoracic level, 10 ml 0.25% bupivacaine before incision, PCEA with bupivacaine 0.125%+ 0.5 µg/ml sufentanil (rate 5–7 ml/hour, 2 ml bolus on demand, lockout time after bolus 20 min) for 96 hours postoperatively | GA (132) | Retrospective cohort study | Open colon cancer surgery |
| Myles 2011 [56] | 1995.7-2001.5 | 9.0-14.8y | 7 | 503 | Various abdominal malignancies | GA+ EDA (230): initiated before surgery and lasted 74h after surgery | GA+PCIA (216) | Previous RCT^2^ | Major abdominal surgery |
| Orriach 2020 [15] | NA | 1005d^1^ | 6 | 100 | Bladder Cancer | GA+EDA (50): at L2-L3 level, 2% lidocaine intraoperatively, 0.2% ropivacaine in the postoperative period to 48 h | GA+ morphic chloride postoperatively (50) | Prospective study | Radical cystectomy |
| Pei 2020 [57] | 1984.2- 2010.2 | 70m^1^ | 8 | 194 | Gastric cancer without metastases | EDA (97): perioperatively | GA (97) | Retrospective cohort study | Total or subtotal gastrectomy+ lymphadenectomies |
| Rahman 2022 [58] | NA | 3y | 4 | 451 | Gastric cancer | GA+EDA (75): at L1-2, 0.7% ropivacaine in 6 – 8 ml, ropivacaine (0.7%, 5ml) every 1 h during surgery, PCA with morphic postoperatively | GA +PCIA (75) | Retrospective cohort study | Radical resection of gastric cancer |
| Roiss 2014 [59] | 2002-2007 | 84m^1^ | 7 | 4772 | Prostate cancer | GA+SPA (3047): at L3-4 or L4-5, single injection (3-4 ml bupivacaine 0.5% isobar + sufentanil 5 mg before surgery | GA (1725) | Retrospective cohort study | Radical retropubic prostatectomy |
| Scavonetto 2014 [60] | 1991.1-2005.12 | 8.6 y | 7 | 3284 | Prostate cancer | GA+SPA/EDA (1642): SPA single injection12–15 mg of 0.5% bupivacaine combined with either morphine 0.3–0.6 mg or hydromorphone 50–60 mg before surgery; EDA: loading dose 0.5% or 0.75% bupivacaine or 2% lidocaine+ fentanyl 100 mg before surgery and fentanyl 10 ug/ml at a rate of 7–12 ml/h for 48/72h after surgery | GA (1642) | Retrospective cohort study | Radical retropubic prostatectomy |
| Shin 2017 [61] | 2005.11-2010.12 | 5y/ 53.3 ± 21.7 m(mean) | 9 | 3799 | Gastric cancer | GA+PCEA (3245): 0.15% ropivacaine and mixed with fentanyl at dose of 2–4 μg/mL at rate of 4–5 mL/h, bolus doses of 1–2 mL, and lock-out times of 15 min | GA +PCIA (374) | Retrospective cohort study | Gastrectomy |
| Sprung 2014 [62] | 1991.1.1-1996.1.31 | 16.2y (median) | 8 | 972 | Prostate Cancer | EDA (486): at lumbar level, 0.5%–0.75% bupivacaine + 50–100 μg fentanyl before surgery, additional local anesthetic administered when needed intraoperatively, 10 μg/mL fentanyl at rate of 7 and 10 mL/h for 1 to 3 days postoperatively | GA (486) + oral opioid postoperatively | Retrospective cohort study | Radical prostatectomy |
| Tai 2018 [63] | 2005.1.1-2014.12. 31 | 18.3 m (median) | 7 | 999 | Stage IV colorectal cancer | GA+EDA (165): a low thoracic level (e.g., T10–T12), lidocaine 1% or 2% with or without fentanyl 50 μg given before surgical incision, continuous infusion bupivacaine 0.25% or 0.5% and fentanyl 5 μg/ml at a rate of 5–10 ml/h, epidural infusion of diluted local anesthetic solution for 48 to 72h postoperatively | GA +PCIA (834) | Retrospective cohort study | Primary tumor resection |
| Tseng 2014 [64] | 1999.1-2005.12 | 10 y | 7 | 1964 | Prostate Cancer | SPA (1166): perioperatively | GA (798) | Retrospective cohort study | Radical Prostatectomy |
| Tseng 2018 [65] | 2005.1–2013.12 | 156m^1^ | 7 | 648 | Advanced ovarian  cancer | GA+EDA (435): 0.05% bupivacaine with or without hydromorphone, fentanyl, or morphine, continuous dose of 3 mL/h and a patient-initiated demand dose of 3 mL every 30 min started either intraoperatively or immediately postoperatively | GA+PCIA (213) | Retrospective cohort study | Primary debulking surgery |
| Tsui 2010 [66] | 2000-2001 | 5 y | 7 | 99 | Prostate cancer | GA+EDA (49): at thoracic or lumbar level, loading dose of 0.5% ropivacaine and continuous infusion of 0.2% ropivacaine with fentanyl 2 μg/ml intraoperatively | GA (50) | Previous RCT^2^ | Radical prostatectomy |
| Vogelaar 2015 [67] | 1995.1-2003.12 | 53 m | 8 | 588 | Colon cancer | GA+EDA (399): perioperatively | GA+PCIA (189) | Retrospective cohort study | Colorectal cancer surgery |
| Wang 2016 [68] | 2006.8 - 2010.12 | 8 y | 8 | 373 | Gastric cancer | GA+EDA (157): 0.25% levobupivacaine or ropivacaine via epidural catheter intraoperatively | GA (116) | Retrospective cohort study | Gastric cancer surgery |
| Wang 2017 [69] | 2008-2012 | 3-7y | 7 | 4218 | Gastric cancer | EDA (1362): at T8-T9, 3ml bolus of 1.33% lidocaine before induction, continue infusion with 0.5% levobupivacaine or ropivacaine during surgery, PCEA with 0.125% levobupivacaine combined with 2 μg /ml fentanyl or 0.2% ropivacaine combined with 2 μg /ml fentanyl for 72 to 120 h postoperatively | GA +PCIA (2856) | Retrospective cohort study | Gastric cancer resection |
| Weingarten 2016 [70] | 1998.1.1-2007.12.31 | 5.9 y | 9 | 1373 | Bladder cancer | GA+SA (207): SA single dose with morphine (0.3-0.6 mg) or hydromorphone (50-60 lg) before induction | GA (1166) | Retrospective cohort study | Radical cystectomy |
| Wu 2021 [16] | 2005-2014 | 2-11y/ 46.1m(median) | 8 | 1282 | Stage I–III rectal cancer | GA+EDA (237):at T10- T12, lidocaine 60–100 mg before incision, continued with bupivacaine 0.25% or 0.5% with or without fentanyl 1-2 µg/ml at rate of 5-10 ml for 3d postoperatively | GA (1045) | Retrospective cohort study | Rectal cancer resection |
| Wuethrich 2010 [71] | 1994.1-2000.12 | EDA 11.9y;  GA 8.5y (median) | 7 | 261 | Prostate cancer | GA+EDA (103): at T10–T11 or T11–T12, 0.25% bupivacaine at a rate of 8–10 ml/h intraoperatively, 0.1% bupivacaine combined with 2 μg /ml epinephrine and 2μg /ml fentanyl at rate of 8–15 ml/h for at least 48 h + 1,000 mg paracetamol every 6 h postoperatively | GA+ ketorolac IV postoperatively (158) | Retrospective cohort study | Radical prostatectomy |
| Wuethrich 2013 [72] | 1994.1-2000.12 | 14y (median) | 8 | 148 | Locally advanced  prostate cancer | GA+EDA (67): 0.25% bupivacaine at a rate of 8-10ml/h intraoperatively, 0.1% bupivacaine +2 µg/ml epinephrine and 2 µg/ml fentanyl 8 to 15 ml/h for at least 48 h postoperatively+ 1000mg paracetamol IV every 6h | GA+ ketorolac IV postoperatively (81) | Retrospective cohort study | Retropubic radical prostatectomy |
| Yen 2021 [17] | 2011.1.1-2017.12.31 | 50 m (median) | 8 | 725 | Renal cell carcinoma | GA+EDA (145): at the lower thoracic or high lumbar region (T10–L2), loading dose 1–1.5% lidocaine with or without fentanyl 50 µg before surgical incision; 0.25% bupivacaine 5–10 ml/h intraoperatively; 0.0625% bupivacaine for 48–72 h postoperatively | GA+ PCIA (580) | Retrospective cohort study | Curative surgery for renal cell carcinoma |
| Zhong 2019 [18] | 2015.12-2017.6 | 1y | 6 | 298 | Ovarian cancer | SA+EDA (158):SA at L3-L4 and EDA at T10-T11, 1.5% lidocaine 3 ml +0.16% amethocaine and adrenalin (200,000:1) before surgery; 0.25% ropivacaine 5 ml before the end of surgery, 0.15% ropivacaine + 0.6 µg/kg fentanyl 2ml/h postoperatively | GA+ fentanyl postoperatively (140) | Retrospective cohort study | Extensive total hysterectomy and pelvic lymphotomy |
| Zhu 2017 [19] | 2010.1- 2012.1 | 3 y | High risk ^4^ | 144 | Gallbladder cancer | GA+EDA (72): at T10-T11, initial dose 5 mL of 0.5% levobupivacaine+ additional 5–10 mL LA before surgery | GA (72) | Randomized controlled trial | Cholecystectomy/ radical cholecystectomy |
| Zimmitti 2015[73] | 2006.1- 2011.10 | 5 y | 7 | 510 | Colorectal liver metastases | GA+EDA (390): at T7-T10, 0.075 % bupivacaine+ 5 μg /cc of hydromorphone intraoperatively and continued postoperatively until full liquid diet | GA+PCIA (120) | Retrospective cohort study | Liver resections |

^1^ The longest follow up time was obtained from Kaplan-Meier survival curve figure.

^2^ *Post hoc* analyses of data from previous RCT study was designed initially to investigate the association between RA and other outcomes.

^3^ 67.9% of procedures were major abdominal cancer surgeries.

^4^The risk of bias of RCTs or prospective non-RCTs was assessed by the Cochrane Collaboration’s tool.

EDA, epidural anesthesia, and analgesia; GA, general anesthesia; HR, hazard ratio; IV, intravenous; LA, local anesthetics; NA, not available; NOS, Newcastle-Ottawa Scale; PCEA, patient-controlled epidural analgesia; PCIA, patient-controlled intravenous analgesia; RAA, regional anesthesia and analgesia; RCT, randomized controlled trial; SPA, spinal anesthesia

**e-table 3: Cohort studies assessed by Newcastle-Ottawa Scale (NOS)**

| Clinical trials | Score | Representativeness of the exposed cohort | Selection of the non exposed cohort | Ascertainment of exposure | Demonstration that outcome of interest was not present at start of study | Comparability of cohorts on the basis of the design or analysis | | Assessment of outcome | Was follow-up long enough for outcomes to occur | Adequacy of follow up of cohorts |
| --- | --- | --- | --- | --- | --- | --- | --- | --- | --- | --- |
| Alexander 2021 | 7 |  | * | * | * | * |  | * | * | * |
| Anic 2022 | 7 |  | * | * | * | * |  | * | * | * |
| Balakrishnan 2022 | 7 |  | * | * | * | * |  | * | * | * |
| Biki 2008 | 6 |  | * | * | * |  |  | * | * | * |
| Binczak M 2013 | 7 |  | * | * | * | * |  | * | * | * |
| Cao 2014 | 7 |  | * | * | * | * |  | * | * | * |
| Capmas 2012 | 6 |  | * | * | * |  |  | * | * | * |
| Chang 2019 | 8 |  | * | * | * | * | * | * | * | * |
| Chipollini 2018 | 7 |  | * | * | * | * | * | * | * |  |
| Christopherson 2008 | 7 |  | * | * | * | * |  | * | * | * |
| Cummings 2012 | 8 |  | * | * | * | * | * | * | * | * |
| Cummings III 2014 | 8 |  | * | * | * | * | * | * | * | * |
| Day 2012 | 7 |  | * | * | * | * | * |  | * | * |
| de Oliveira 2011 | 6 |  | * | * | * |  |  | * | * | * |
| Doiron 2016 | 6 | * | * | * | * |  |  | * | * |  |
| Ehdaie 2014 | 9 | * | * | * | * | * | * | * | * | * |
| Elias 2015 | 7 |  | * | * | * |  | * | * | * | * |
| Gao 2019 | 6 |  | * | * | * |  |  | * | * | * |
| Garland 2018 | 7 |  | * | * | * | * |  | * | * | * |
| Gottschalk 2010 | 7 |  | * | * | * | * |  | * | * | * |
| Gupta 2011 | 6 |  | * | * | * |  |  | * | * | * |
| Hasselager 2022 | 9 | * | * | * | * | * | * | * | * | * |
| Hiller 2014 | 5 |  | * | * | * |  |  | * |  | * |
| Holler 2013 | 5 |  | * | * | * |  |  | * | * |  |
| Lacassie 2013 | 9 | * | * | * | * | * | * | * | * | * |
| Lin 2011 | 6 |  | * | * | * |  |  | * | * | * |
| Lin 2021 | 8 |  | * | * | * | * | * | * | * | * |
| Liu 2021 | 4 |  | * |  | * | * |  | * |  |  |
| Lorimier 2018 | 8 |  | * | * | * | * | * | * | * | * |
| Moormann 2022 | 7 |  | * | * | * | * |  | * | * | * |
| Myles 2011 | 7 | * | * | * | * |  |  | * | * | * |
| Orriach 2020 | 6 |  | * | * | * | * | * | * |  |  |
| Pei 2020 | 8 |  | * | * | * | * | * | * | * | * |
| Rahman 2022 | 4 |  | * |  | * | * |  |  | * |  |
| Roiss 2014 | 7 |  | * | * | * | * | * | * | * |  |
| Scavonetto 2014 | 7 |  | * | * | * | * |  | * | * | * |
| Shin 2017 | 9 | * | * | * | * | * | * | * | * | * |
| Sprung 2014 | 8 |  | * | * | * | * | * | * | * | * |
| Tai 2018 | 7 |  | * | * | * |  | * | * | * | * |
| Tseng 2014 | 7 | * | * | * | * |  |  | * | * | * |
| Tseng 2018 | 7 | * | * | * | * |  |  | * | * | * |
| Tsui 2010 | 7 |  | * | * | * | * |  | * | * | * |
| Vogelaar 2015 | 8 |  | * | * | * | * | * | * | * | * |
| Wang 2016 | 8 |  | * | * | * | * | * | * | * | * |
| Wang 2017 | 7 |  | * | * | * | * |  | * | * | * |
| Weingarten 2016 | 9 | * | * | * | * | * | * | * | * | * |
| Wu 2021 | 8 |  | * | * | * | * | * | * | * | * |
| Wuethrich 2010 | 7 |  | * | * | * | * | * | * | * |  |
| Wuethrich 2013 | 7 |  | * | * | * | * |  | * | * | * |
| Yen 2021 | 8 |  | * | * | * | * | * | * | * | * |
| Zhong 2019 | 6 |  | * | * | * | * |  | * | * |  |
| Zimmitti 2015 | 7 |  | * | * | * | * |  | * | * | * |

**e-Table 4:** **RCTs assessed by Cochrane Collaboration’s tool. RCTS, randomized controlled trials.**

| Clinical trials | Random sequence generation (selection bias) | Allocation concealment (selection bias) | Blinding of participants and personnel (performance bias) | Blinding of outcome assessment (detection bias) | Incomplete outcome data (attrition bias) | Selective reporting (reporting bias) | Other bias | Risk of bias |
| --- | --- | --- | --- | --- | --- | --- | --- | --- |
| Du 2019 | low | low | low | low | low | low | low | low |
| Falk 2021 | low | low | low | low | low | low | low | low |
| Zhu 2017 | high | high | high | low | low | low | low | high |

**e-Table 5: Weighted Kappa Measurements to Assess Agreement Between Reviewers in Rating Quality of Methodology of Included Trials**

**Risk of bias for randomized controlled trial.**

|  | κ |
| --- | --- |
| Random sequence generation | 1.00 |
| Allocation concealment | 0.95 |
| Performance bias | 0.90 |
| Detection bias | 0.83 |
| Attrition bias | 0.92 |
| Reporting bias | 0.68 |
| Other bias | 0.81 |
| Overall bias | 0.87 |

**NOS scores for observational study**

|  | κ |
| --- | --- |
| Representativeness of the exposed cohort |  |
| Truly representative of the average incidence of cancer in the community | 0.80 |
| Somewhat representative of the average incidence of cancer in the community | 0.88 |
| Selection of the nonexposed cohort |  |
| Drawn from the same community as the exposed cohort | 0.78 |
| Ascertainment of exposure | 0.85 |
| Demonstration that outcome of interest was not present at start of study | 0.83 |
| Comparability of cohorts on the basis of the design or analysis |  |
| Study controls for age | 0.92 |
| Study controls for any additional factors: tumor stage, transfusion, blood loss | 0.94 |
| Assessment of outcome |  |
| Independent blind assessment | 0.96 |
| Record linkage | 0.92 |
| Was follow-up long enough for outcomes to occur |  |
| Complete follow up - all subjects accounted for | 1.00 |
| Subjects lost to follow up unlikely to introduce bias - small number lost follow up rate > 70 % | 0.85 |
| Adequacy of follow up of cohorts | 0.98 |

**e-Table 6：Grade quality of evidence for cancer recurrence**

**Author(s):** Lin Lu，Yanxia Sun, Yi Ren, Siwen Zhao and Zhen Hua
**Date:** 2022-12-05
**Question:** Should perioperative regional anesthesia be used for reducing cancer recurrence after abdominal oncological surgery?
**Settings:**
**Bibliography:**

| **Quality assessment** | | | | | | | **No of patients** | | **Effect** | | **Quality** | **Importance** |  |
| --- | --- | --- | --- | --- | --- | --- | --- | --- | --- | --- | --- | --- | --- |
|  |  |  |  |  |  |  |  |  |  |  |  |  |  |
| **No of studies** | **Design** | **Risk of bias** | **Inconsistency** | **Indirectness** | **Imprecision** | **Other considerations** | **Perioperative regional anesthesia** | **Control** | **Relative (95% CI)** | **Absolute** |  |  |  |
| **Cancer recurrence (follow-up 1-17 years)** | | | | | | | | | | | | |  |
| 36 | observational studies^1^ | serious^1^ | serious^2^ | no serious indirectness | no serious imprecision | none^3^ | 5732/30389  (18.9%) | 9679/51431  (18.8%) | HR 0.98 (0.91 to 1.05) | 3 fewer per 1000 (from 15 more to 8 more) | ÅOOO VERY LOW | CRITICAL |  |
|  |  |  |  |  |  |  |  | 18.8% |  | 3 fewer per 1000 (from 15 fewer to 8 more) |  |  |  |
| **cancer recurrence (follow-up 1-5 years; assessed with: cancer recurrence)** | | | | | | | | | | | | |  |
| 2 | randomized trials | no serious risk of bias | no serious inconsistency | no serious indirectness | no serious imprecision | reporting bias^4^ | 470/942  (49.9%) | 495/950  (52.1%) | HR 0.87 (0.72 to 1.04) | 48 fewer per 1000 (from 110 fewer to 14 more) | ÅÅÅO MODERATE | CRITICAL |  |
|  |  |  |  |  |  |  |  | 18.8% |  | 22 fewer per 1000 (from 49 fewer to 7 more) |  |  |  |

^1^ all included study were retrospective design with long follow-up. some of included studies were large sample size, some were reanalysis from previous RCT
^2^ the p value for heterogeneity less than 0.05 and I^2^ 52.3%. Heterogeneity could be partly explained by type of cancer, or surgical techniques
^3^ some type of cancer for example ovarian cancer may benefited from regional anesthesia
^4^ a limited number of included studies may induce potential publication bias

**e-Table 7: Meta- regression analyses for overall survival and cancer recurrence**

|  |  |  |
| --- | --- | --- |
|  | Overall survival loghr(SE) | Cancer recurrence loghr(SE) |
| NOS | 0.0264 | -0.0517 |
|  | (0.0470) | (0.0529) |
| Bladder cancer | 0.447 | 0.293 |
|  | (0.347) | (0.315) |
| Colorectal cancer | 0.110 | -0.194 |
|  | (0.326) | (0.257) |
| Gastric cancer | 0.0955 | -0.116 |
|  | (0.333) | (0.324) |
| Hepatocellular carcinoma | 0.465 | -0.302 |
|  | (0.360) | (0.270) |
| Pancreatic cancer | 0.0704 |  |
|  | (0.343) |  |
| Ovarian cancer | 0.368 | -0.126 |
|  | (0.390) | (0.261) |
| Prostate cancer | 0.205 | -0.552 |
|  | (0.346) | (0.330) |
| Various abdominal malignancies | -0.0731 | -0.260 |
|  | (0.407) | (0.302) |
| sample size | 0.00000396 | 0.00000357 |
|  | (0.00000509) | (0.00000464) |
|  |  |  |
| _cons | -0.556 | 0.517 |
|  | (0.456) | (0.489) |
| *N* | 41 | 38 |
| % residual variation due to heterogeneity | 61.9% | 48.68% |
| Proportion of between-study variance explained | 9.17% | 24.63% |

**e-Table 8：Grade quality for overall survival**

**Author(s):** Lin Lu, Yanxia Sun, Yi Ren, Siwen Zhao and Zhen Hua
**Date:** 2022-12-05
**Question:** Should perioperative regional anesthesia be used for reducing mortality after abdominal oncological surgery ?^1^
**Settings:**
**Bibliography:**

| **Quality assessment** | | | | | | | **No of patients** | | **Effect** | | **Quality** | **Importance** |  |
| --- | --- | --- | --- | --- | --- | --- | --- | --- | --- | --- | --- | --- | --- |
|  |  |  |  |  |  |  |  |  |  |  |  |  |  |
| **No of studies** | **Design** | **Risk of bias** | **Inconsistency** | **Indirectness** | **Imprecision** | **Other considerations** | **Perioperative regional anesthesia** | **Control** | **Relative (95% CI)** | **Absolute** |  |  |  |
| **mortality (follow-up 1-10 years; assessed with: overall survival)** | | | | | | | | | | | | |  |
| 37 | observational studies | serious^2^ | serious^3^ | no serious indirectness | no serious imprecision | none^4^ | 10068/32733  (30.8%) | 20299/53048  (38.3%) | HR 0.85 (0.80 to 0.91) | 48 fewer per 1000 (from 30 more to 67 more) | ÅOOO VERY LOW | CRITICAL |  |
|  |  |  |  |  |  |  |  | 38.3% |  | 46 fewer per 1000 (from 27 fewer to 63 fewer) |  |  |  |
| **mortality (follow-up 1-5; assessed with: mortality)** | | | | | | | | | | | | |  |
| 2 | randomized trials | serious^5^ | no serious inconsistency | no serious indirectness | no serious imprecision | reporting bias^6^ | 388/927  (41.9%) | 355/931  (38.1%) | HR 0.91 (0.76 to 1.10) | 27 fewer per 1000 (from 76 fewer to 29 more) | ÅÅOO LOW | CRITICAL |  |
|  |  |  |  |  |  |  |  | 38% |  | 27 fewer per 1000 (from 75 fewer to 29 more) |  |  |  |

^1^ overall survival
^2^ studies were retrospective design with long follow-up. some of them were post hoc analysis data from previous randomized controlled trials performed for others outcomes
^3^ the p value for heterogeneity less than 0.05 and I2 of 60.2%.
^4^ several confounders may influence the main results for example type of cancer, surgical techniques.
^5^ one trial was low risk of bias and the other was high risk of bias
^6^ a limited number of included studies may induce to potential publication bias


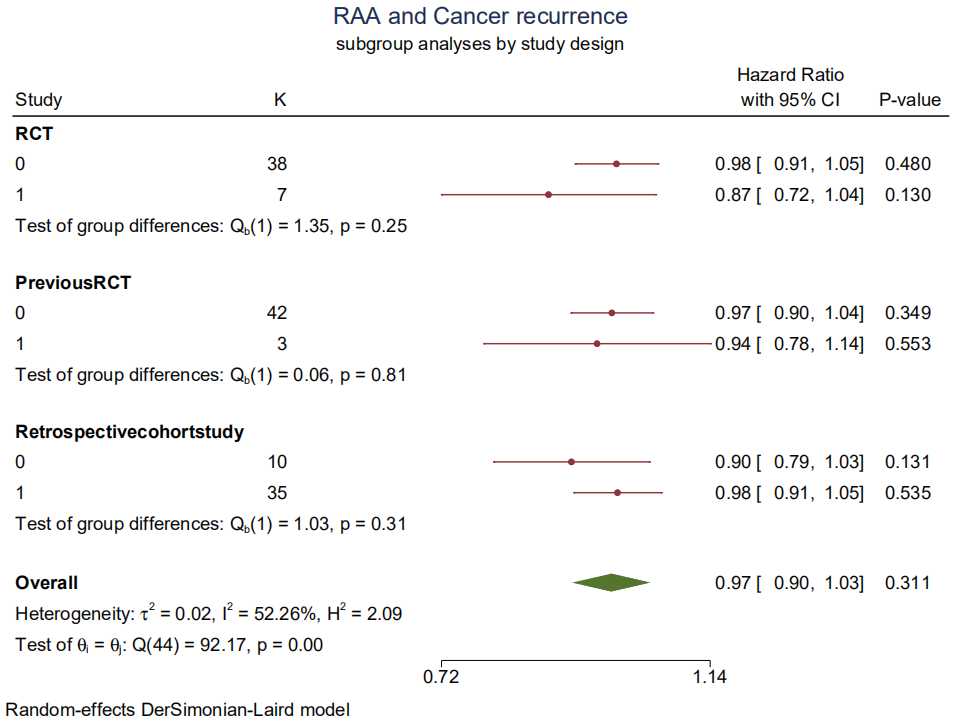


Figure S1: RAA and cancer recurrence: Forrest plots of subgroup analysis based on study design for cancer recurrence. RCT: Randomized controlled trial.


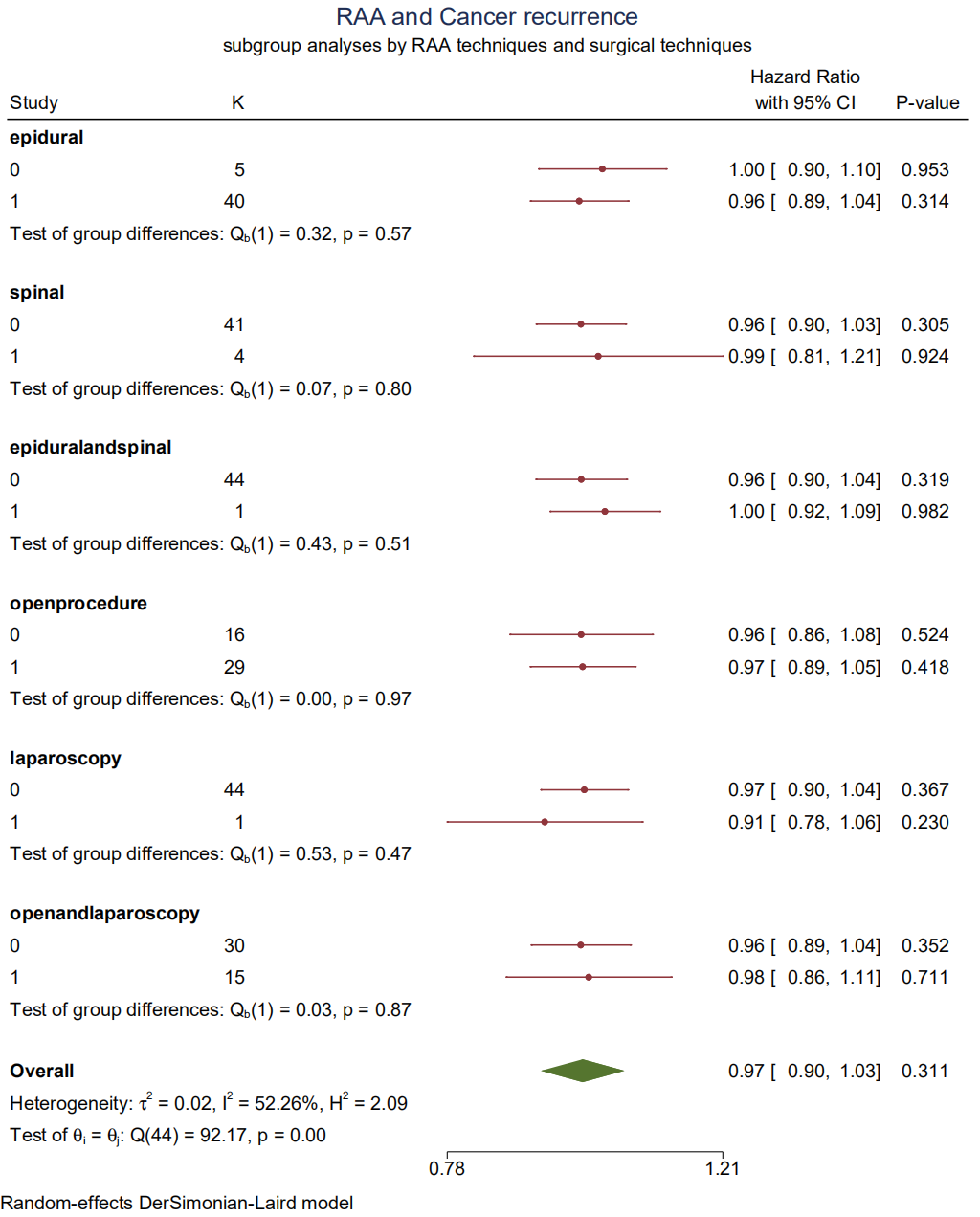


Figure S2: RAA and cancer recurrence: Forrest plots of subgroup analysis based on RAA techniques and surgical techniques.


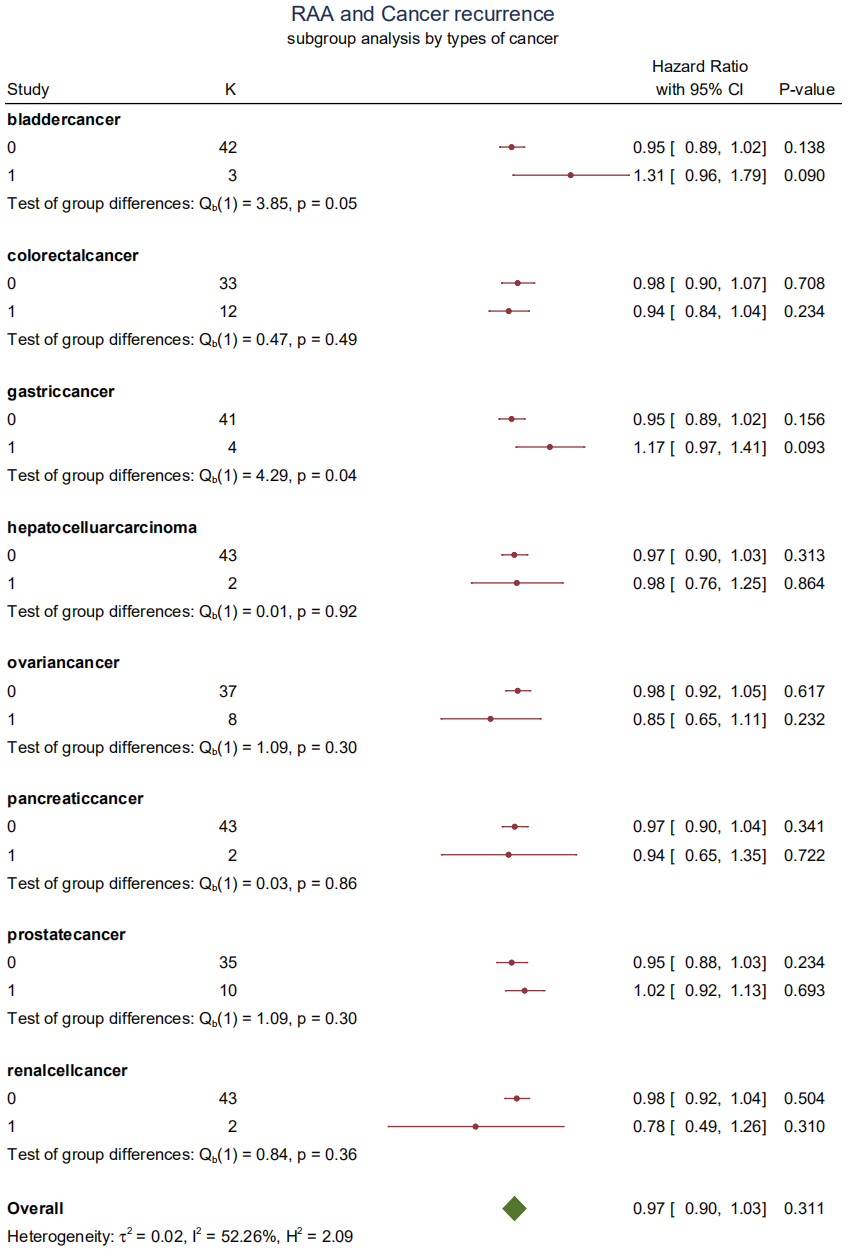


Figure S3: RAA and cancer recurrence: Forrest plots of subgroup analysis based on types of cancer

Figure S4：RAA and cancer recurrence: Leave-one-out meta-analysis


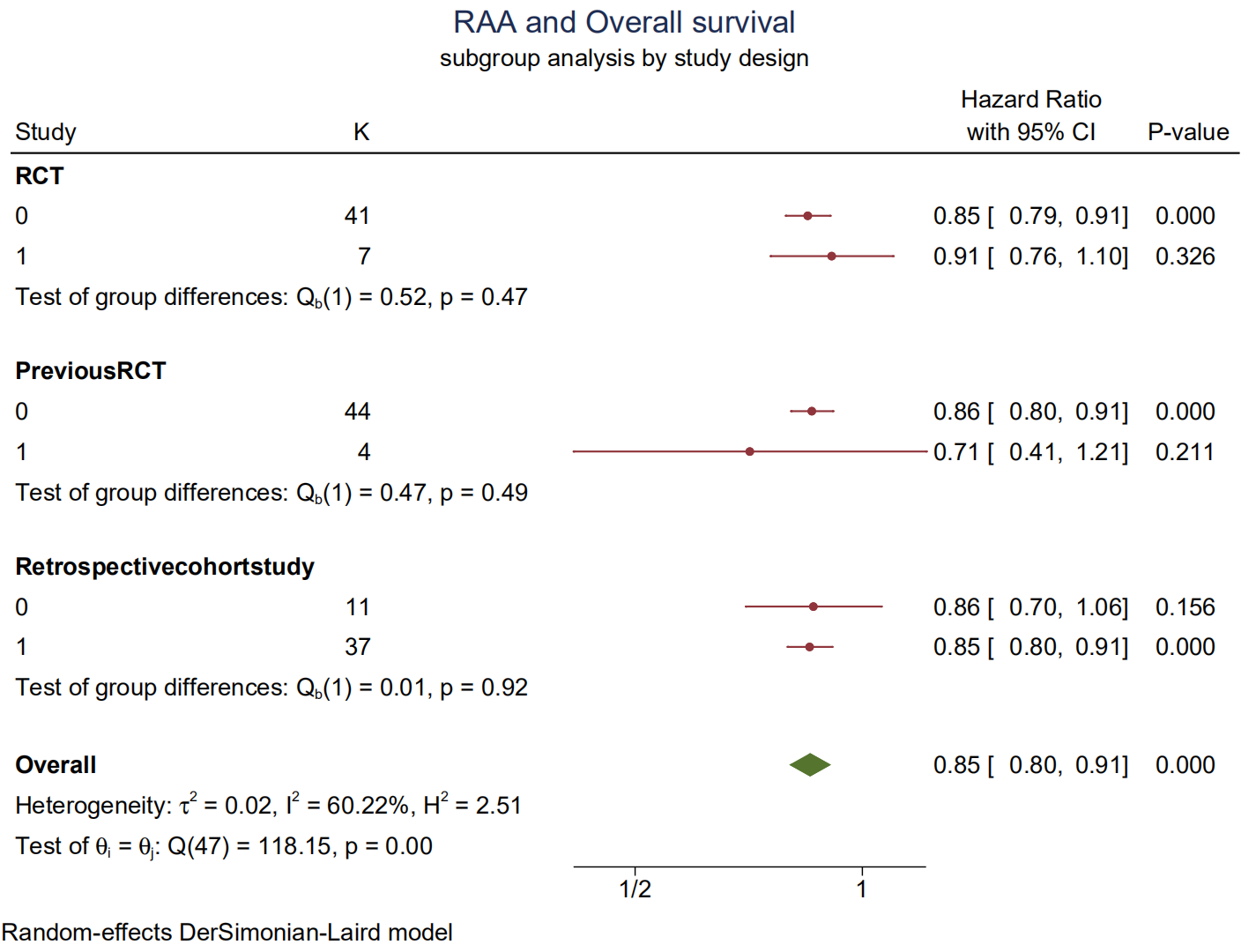


Figure S5: RAA and overall survival: Forrest plots of subgroup analysis based on study design for cancer recurrence. RCT: randomized controlled trial.


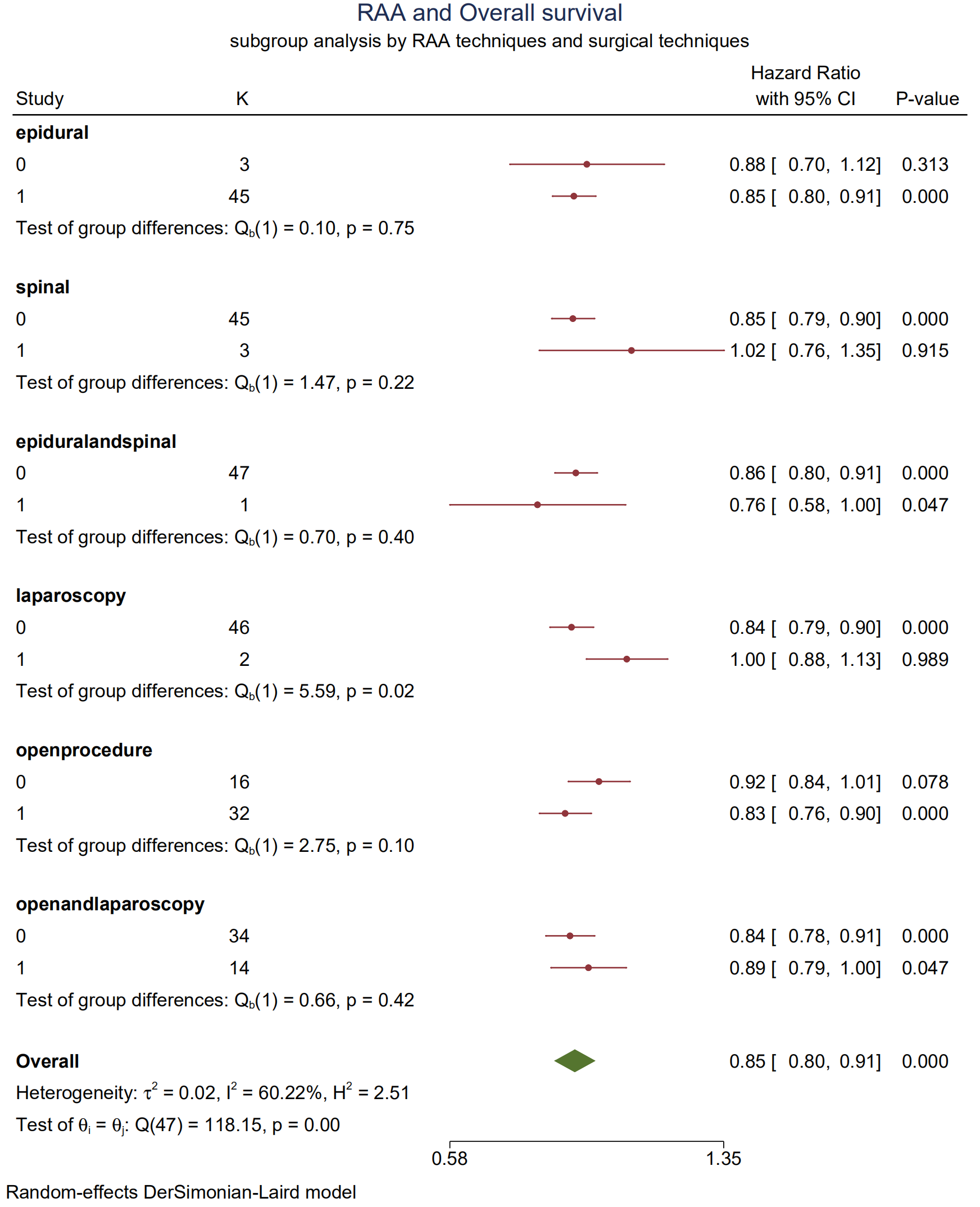


Figure S6: RAA and overall survival: Forrest plots of subgroup analysis based on RAA techniques and surgical techniques.


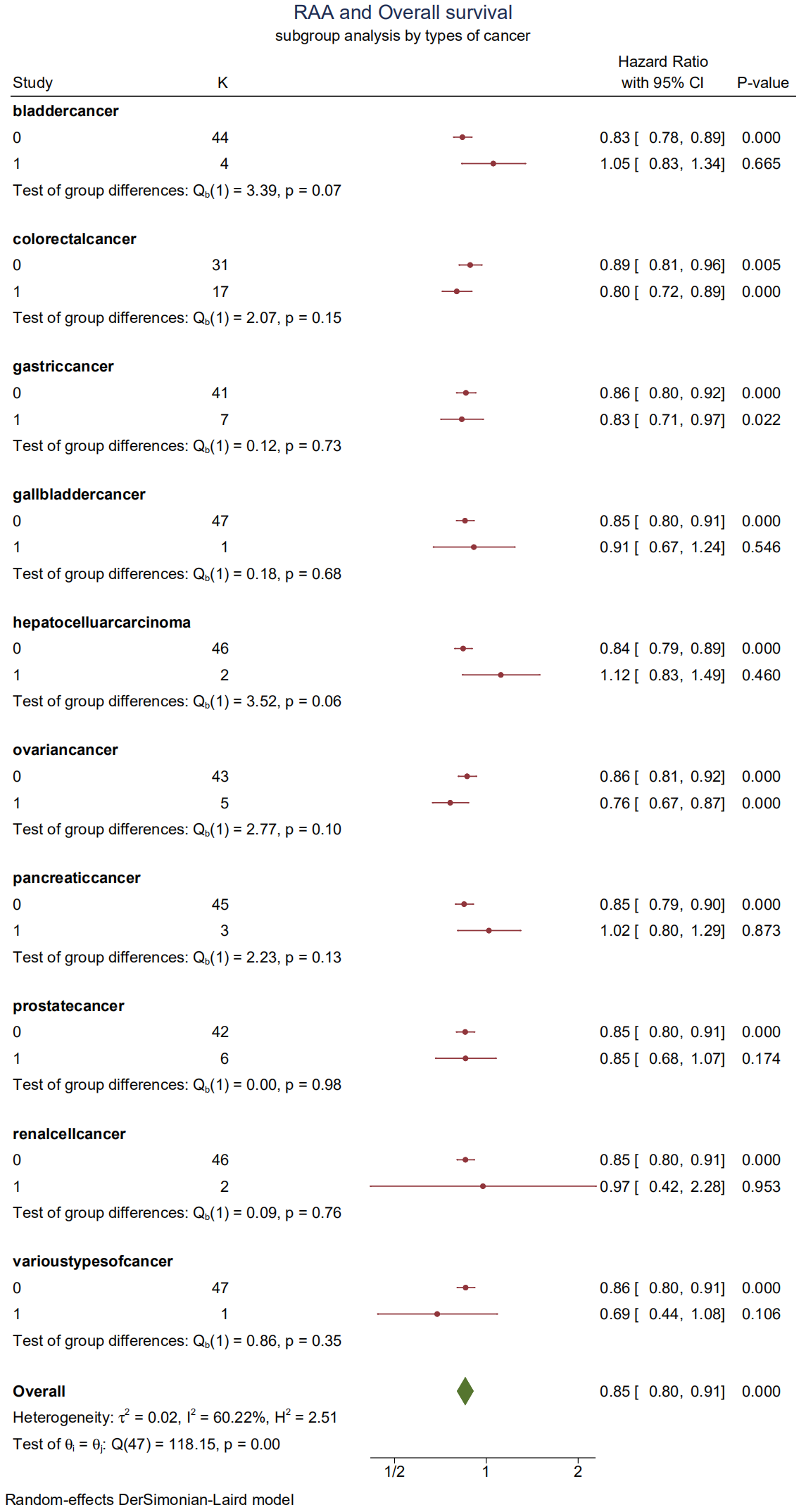


Figure S7: RAA and overall survival: Forrest plots of subgroup analysis based on types of cancer.

Figure S8: RAA and overall survival: Leave-one-out meta-analysis


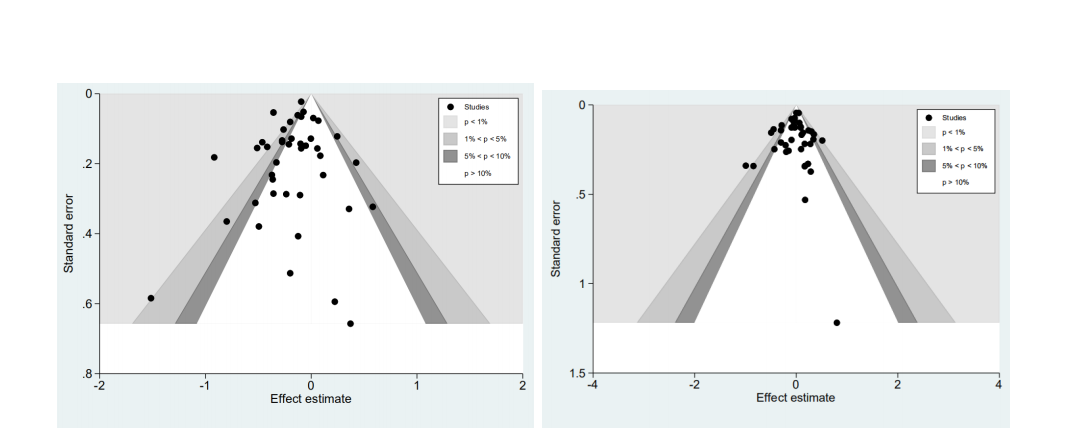


Figure S9: Contour-enhanced funnel plots


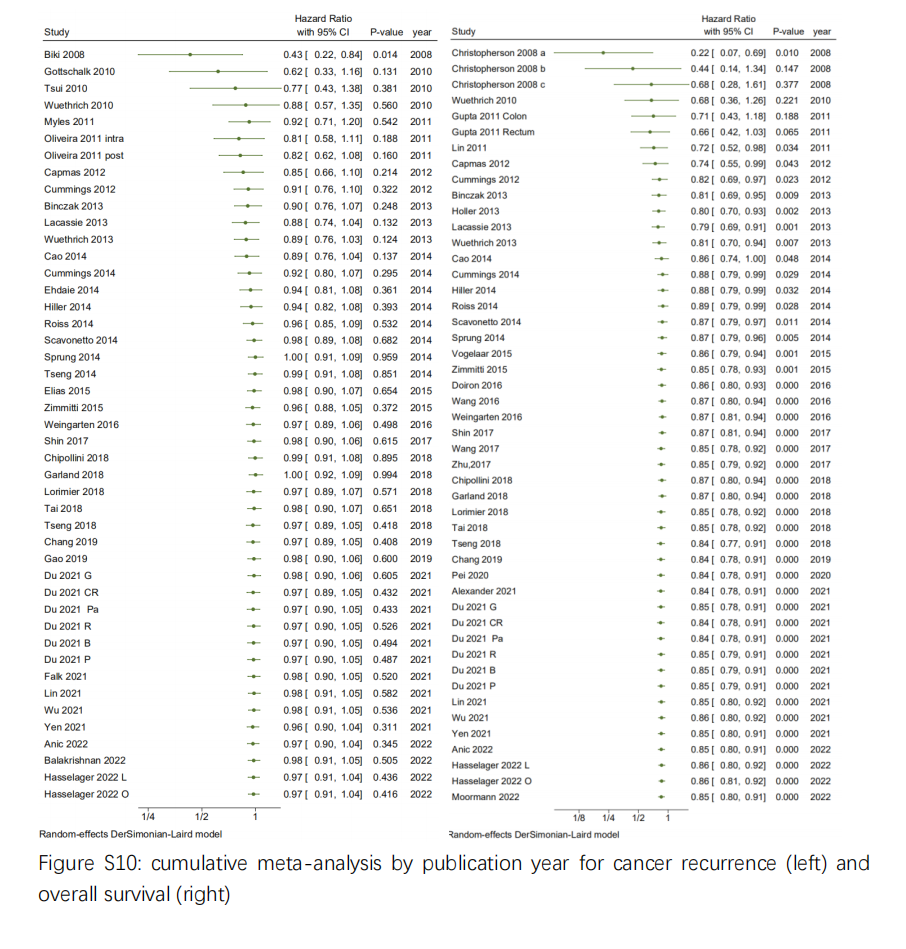


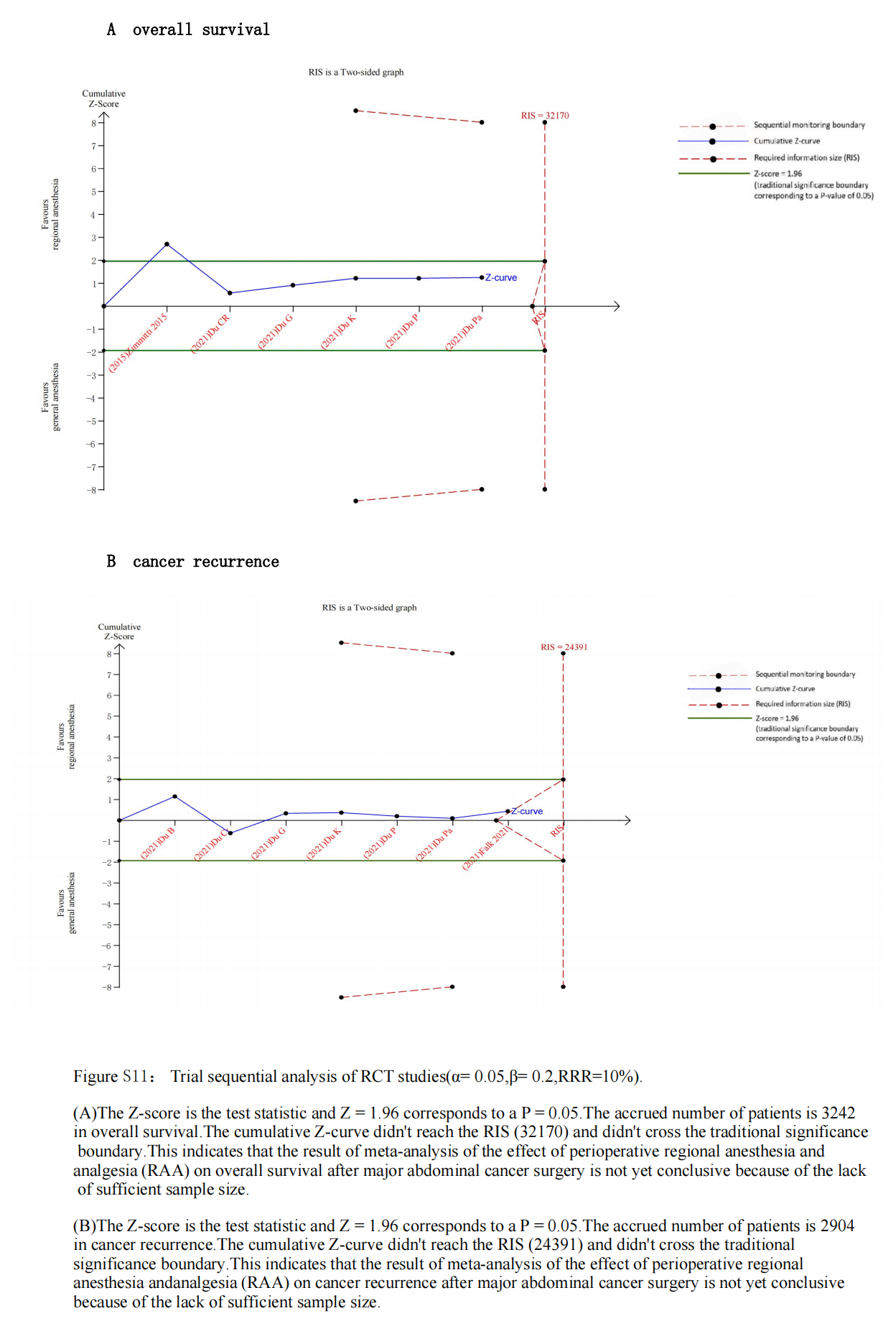

Supplement: Multimedia component 2 [file mmc2.docx]
